# The large soybean (*Glycine max*) WRKY TF family expanded by segmental duplication events and subsequent divergent selection among subgroups

**DOI:** 10.1186/1471-2229-13-148

**Published:** 2013-10-03

**Authors:** Guangjun Yin, Hongliang Xu, Shuyang Xiao, Yajuan Qin, Yaxuan Li, Yueming Yan, Yingkao Hu

**Affiliations:** 1 College of Life Sciences, Capital Normal University, Beijing 100048, China; 2 Biochemistry, Molecular Biology & Biophysics, University of Minnesota, Minneapolis, MN 55455, USA

## Abstract

**Background:**

WRKY genes encode one of the most abundant groups of transcription factors in higher plants, and its members regulate important biological process such as growth, development, and responses to biotic and abiotic stresses. Although the soybean genome sequence has been published, functional studies on soybean genes still lag behind those of other species.

**Results:**

We identified a total of 133 WRKY members in the soybean genome. According to structural features of their encoded proteins and to the phylogenetic tree, the soybean WRKY family could be classified into three groups (groups I, II, and III). A majority of WRKY genes (76.7%; 102 of 133) were segmentally duplicated and 13.5% (18 of 133) of the genes were tandemly duplicated. This pattern was not apparent in *Arabidopsis* or rice. The transcriptome atlas revealed notable differential expression in either transcript abundance or in expression patterns under normal growth conditions, which indicated wide functional divergence in this family. Furthermore, some critical amino acids were detected using DIVERGE v2.0 in specific comparisons, suggesting that these sites have contributed to functional divergence among groups or subgroups. In addition, site model and branch-site model analyses of positive Darwinian selection (PDS) showed that different selection regimes could have affected the evolution of these groups. Sites with high probabilities of having been under PDS were found in groups I, II c, II e, and III. Together, these results contribute to a detailed understanding of the molecular evolution of the WRKY gene family in soybean.

**Conclusions:**

In this work, all the WRKY genes, which were generated mainly through segmental duplication, were identified in the soybean genome. Moreover, differential expression and functional divergence of the duplicated WRKY genes were two major features of this family throughout their evolutionary history. Positive selection analysis revealed that the different groups have different evolutionary rates. Together, these results contribute to a detailed understanding of the molecular evolution of the WRKY gene family in soybean.

## Background

The WRKY family is one of the largest transcription factor families in higher plants, but is absent in animals, extending throughout the entire green lineage. Recently, several WRKY genes were identified from non-plant eukaryotes, including *Dictyostelium discoideum*, a slime mold closely related to the animal and fungi lineages, and the green alga *Chlamydomonas reinhardtii*, an early branch of plants. This suggests that WRKY genes may have had an early origin in lower eukaryotes, which have since greatly expanded in plant species [1]. Since the first WRKY protein, SPF1, was cloned from sweet potato [2], more and more WRKY genes have been experimentally identified in various plant species [3-19]. Each WRKY protein in this family contains at least one WRKY domain of approximately 60 amino acids with the conserved amino acid sequence WRKYGQK at its N-terminus and a novel zinc finger motif, C_2_H_2_ (C–X_4–5_–C–X_22–23_–H–X–H) or C_2_HC (C–X_7_–C–X_23_–H–X–C), at the C-terminal region [20]. The WRKYGQK amino acid sequence forms a β-strand that facilitates binding to the promoters of target genes. Usually, the binding site is the W box, which is an element commonly found in the promoters of many stress-related plant genes [21]. WRKY proteins can be categorized into three groups based on their number of WRKY domains and the pattern of their zinc finger motif [22]. The first group contains two WRKY domains (N-terminal and C-terminal), including a C_2_H_2_ motif, whereas the other two groups have only one domain. Group III has a distinct zinc finger motif, C_2_HC rather than the C_2_H_2_ found in other groups. Group II proteins can be further subdivided into groups II a, II b, II c, II d, and II e based on the amino acid motifs contained outside the WRKY domain.

As transcription factors, plant WRKY proteins have been shown to be involved in responses to biotic and abiotic stresses, and in developmental processes [23]. It has been well documented that WRKY proteins play an important role in plant defense against biotic stresses, such as bacterial, fungal, and viral pathogens [24-27]. They are also key components in developmental processes, including embryogenesis [28], senescence [29], dormancy [30], trichome development [31], seed development [32], and some signal transduction processes mediated by plant hormones such as gibberellic acid [33], abscisic acid (ABA) [34], or salicylic acid [35]. Meanwhile, increasing evidence has revealed that WRKY proteins are involved in responses to various abiotic stresses [36]. In *Arabidopsis*, results of a microarray study demonstrated that the expressions of some WRKY transcripts are regulated in response to abiotic stresses, including salinity, drought, and cold [37-39]. In rice, under various abiotic and phytohormone treatments, the expression of WRKY genes showed significant differences [40]. In *Poncirus trifoliate*, a WRKY gene, *Ptr*WRKY2, showed differential responses to cold and drought stresses [41], while in soybean, at least nine WRKY genes were found to be differentially expressed under abiotic stress [42]. Collectively, this evidence indicates that WRKY genes play important roles in various physiological processes in plants.

Soybean is one of the most important economic crops in the world. Genome and transcriptome sequencing of the palaeopolyploid soybean have been completed [43,44]. In the present study, we searched this genome sequence to identify WRKY proteins, and compared the structure of the encoded proteins with those of their putative homologous WRKY genes in *Arabidopsis*. In order to investigate tandem duplication events, soybean chromosome sequence information was applied to map WRKY transcripts to their corresponding genetic loci on chromosomes. A phylogenetic tree was constructed to evaluate evolutionary relationships among WRKY genes in the two plant species. In addition, we analyzed the transcriptome atlas of WRKY genes in different tissues under normal conditions, and found notable differential expression between groups, which indicated broad functional divergence in this family. Positive selection analysis revealed that evolutionary rates differed among the different groups. Moreover, evolutionary patterns of the WRKY gene family were examined in *Arabidopsis,* rice, and soybean. The results indicated that WRKY genes in soybean were duplicated mainly through segmental duplication, which differed from homologous genes in *Arabidopsis* and rice. These results provide useful information for future studies of molecular evolution of the WRKY gene family in soybean.

## Results

### Identification and distribution of the WRKY gene family in soybean

**Figure 1:**
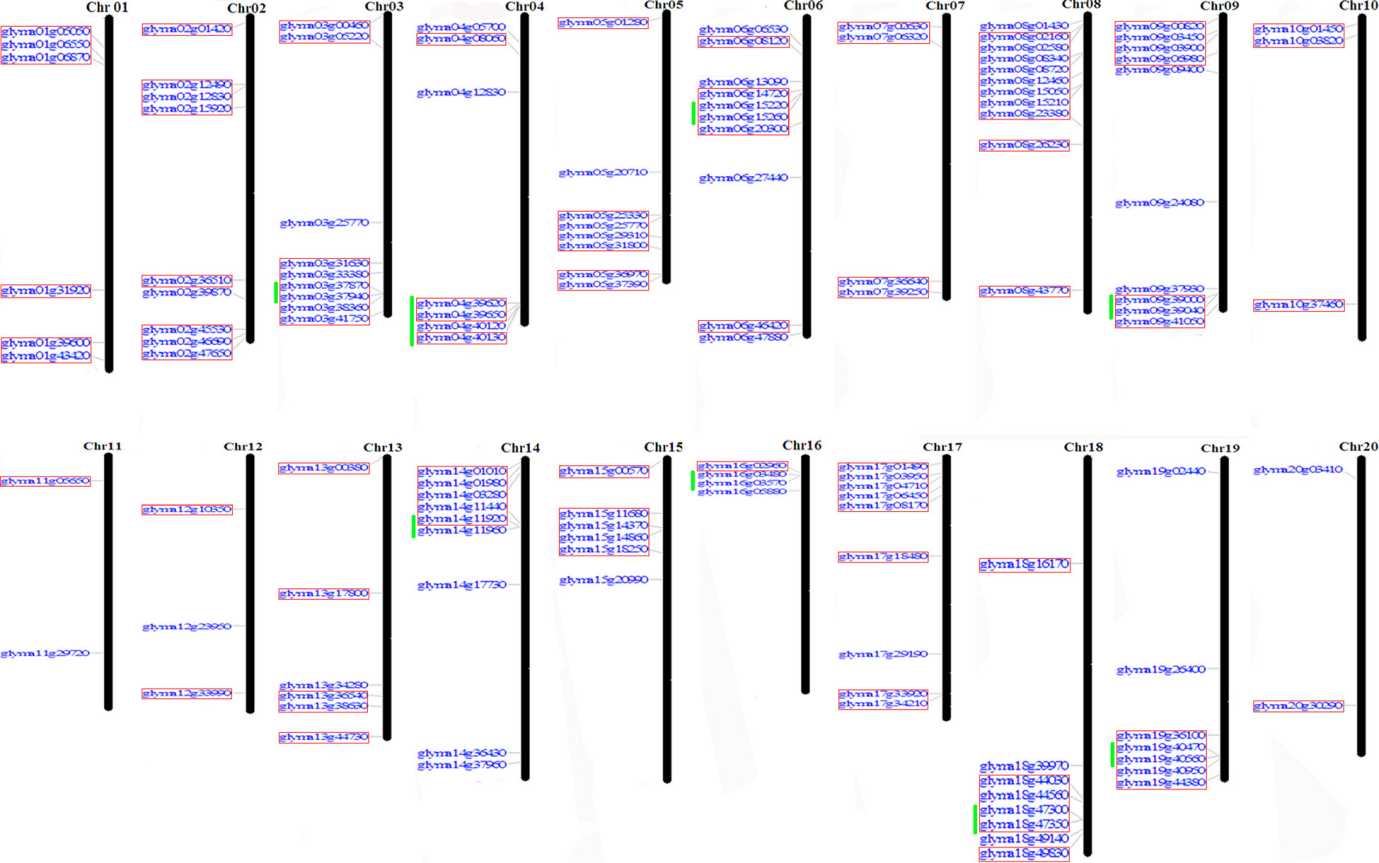
**Chromosome distribution of soybean (*Glycine max*) WRKY genes.** The size of a chromosome is indicated by its relative length. Red outlined boxes represent segmentally duplicated genes. Tandem duplicated genes are indicated with vertical green lines. The location information and chromosome information were obtained from Phytozome. The figure was produced using the MapInspector program.

In plants, the dicot model organism *Arabidopsis* is commonly used to predict the function of a gene in a newly or partially sequenced organism that has a close phylogenetic relationship to *Arabidopsis*, such as soybean. Moreover, there are at least 72 WRKY family members in *Arabidopsis*, and most of these genes have been extensively studied and reported to be involved in many physiological and biochemical processes [20,22]. With the aim of defining the soybean protein-containing WRKY domains, we downloaded the 72 known *Arabidopsis* WRKY protein sequences from the *Arabidopsis* transcription factor database (AtTFDB; http://arabidopsis.med.ohio-state.edu/). In order to examine the structural features of each *At*WRKY, we performed a multiple sequence alignment (data not shown). Two members, *At4g12020* and *At4g30930*, were excluded from the analysis due to incomplete zinc fingers and the lack of WRKY domains. Therefore, 70 *Arabidopsis* WRKY protein sequences were used to BLAST the completed soybean genome sequences for genes that encode proteins containing the WRKY domain. The WRKY domain of each predicted protein was identified by searching against the SMART database. After manually removing overlapping genes, a total of 133 non-redundant genes in the soybean genome were identified as members of the WRKY family (Additional file 1). Among these members, annotation (predicted) of 23 proteins revealed that they have two complete WRKY domains each, which all belong to group I. In addition, physical positions of WRKY genes were obtained from the Phytozome database, and were used to map these genes onto their corresponding soybean chromosomes (Figure 1). Results showed that WRKY genes in soybean could be mapped on all chromosomes, from chromosome 1 to 20. Chromosome 8 had the highest density of WRKY genes with 11 members, while in chromosomes 10, 11, 12, and 20, no more than three WRKY genes could be found, respectively. Examination of the location of each WRKY gene revealed that all *Gm*WRKY genes, except for *Glyma02g39870*, *Glyma03g25770*, *Glyma04g05700*, *Glyma04g12830*, *Glyma05g20710*, *Glyma06g06530*, *Glyma06g13090*, *Glyma06g27440*, *Glyma06g47880*, *Glyma08g01430*, *Glyma09g09400*, *Glyma09g24080*, *Glyma09g37930*, *Glyma11g29720*, *Glyma12g23950*, *Glyma13g34280*, *Glyma14g17330*, *Glyma14g36430*, *Glyma14g37960*, *Glyma15g20990*, *Glyma16g05880*, *Glyma17g29190*, *Glyma18g39970*, *Glyma18g49140*, *Glyma19g02440*, *Glyma19g26400*, and *Glyma20g03410*, originated from segmental duplications (102 of 133) or tandem duplications (18 of 133) (Figure 1). The 27 genes mentioned above might have been produced by retrotransposition instead.

### Multiple sequence alignment and structure analysis

**Figure 2:**
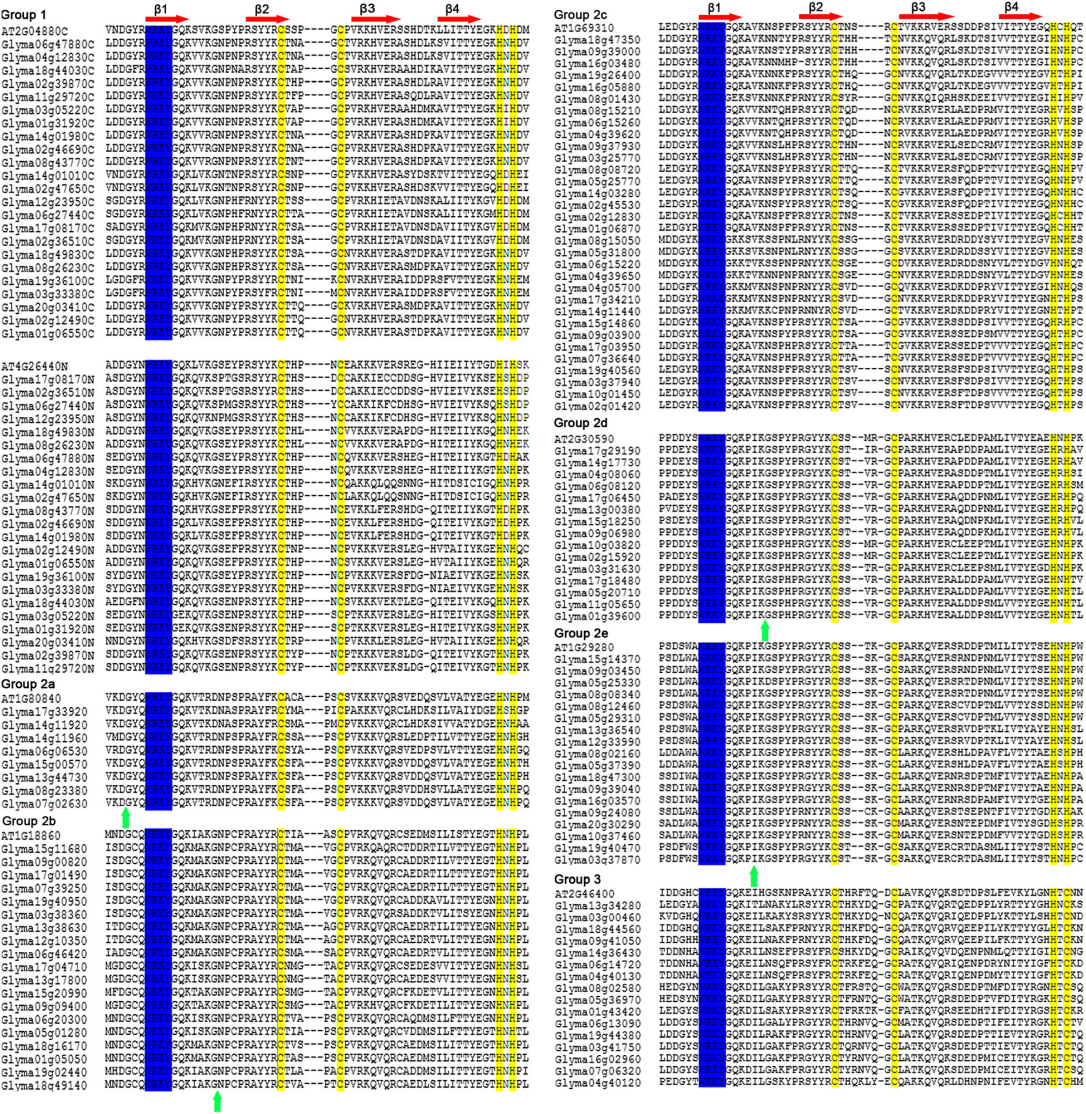
**Alignment of multiple soybean WRKY genes and selected*At*WRKY domain amino acid sequences.** Alignment was performed using Clustal W. The suffixes ‘N’ or ‘C’ denote the N-terminal and C-terminal WRKY domains from Group I WRKY proteins, respectively. The amino acids forming the zinc-finger motif are highlighted in yellow. The conserved WRKY amino acid signature is highlighted in blue. The four β-strands are shown in red. The position of a conserved intron is indicated by an arrowhead.

The phylogenetic relationship of *Gm*WRKY proteins was examined by multiple sequence alignment of their WRKY domains, which span across approximately 60 amino acids (Figure 2). A comparison with soybean WRKY domains and several homologous *Arabidopsis* proteins resulted in a separation of the different groups and subgroups. For each group or subgroup, one *Arabidopsis* protein was selected randomly, which included *At2g04880C*, with only one C-terminal WRKY domain, *At4g26440N*, with only one N-terminal WRKY domain, *At1g80840*, *At1g18860*, *At1g69310*, *At2g30590*, *At1g29280*, and *At2g46400*. As shown in Figure 2, the sequences of soybean WRKY were found to be highly conserved.

**Figure 3:**
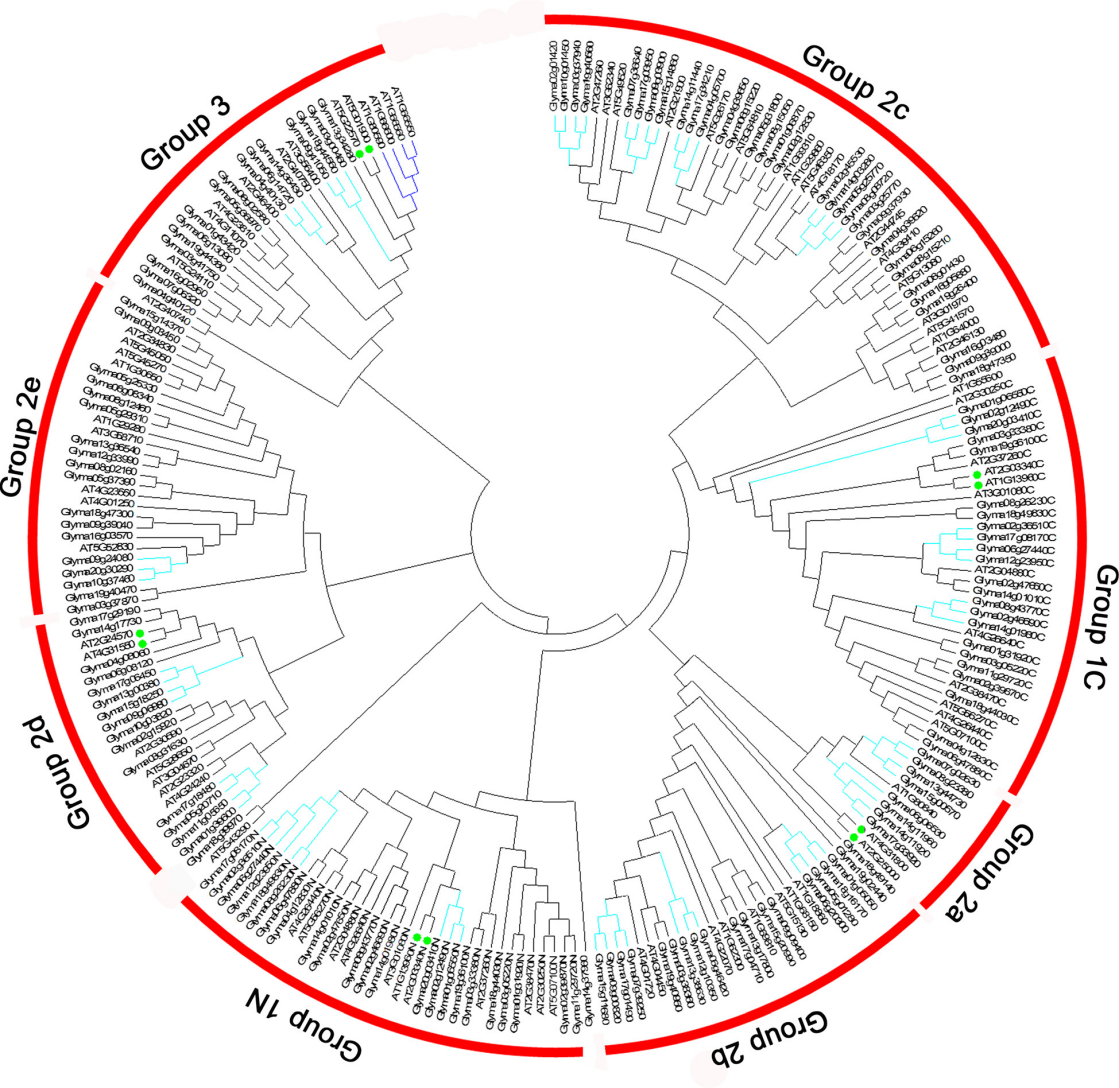
**Phylogenetic tree of WRKY domains among soybean and*Arabidopsis.*** The amino acid sequences of the WRKY domain of soybean and *Arabidopsis* were aligned with Clustal W and the phylogenetic tree was constructed using the maximum likelihood method in MEGA 5.0. Group 1 proteins with the suffix ‘N’ or ‘C’ indicate the N-terminal WRKY domains or the C-terminal WRKY domains, respectively. Genes with similar functions clustered together are indicated by filled green circles. Gene expansion in soybean and *Arabidopsis* are indicated by coloring the subclade with the same color as the leaf label. The red arcs indicate different groups (or subgroups) of WRKY domains.

In order to better separate the groups and examine the evolutionary relationships of the WRKY family in soybean and *Arabidopsis*, an unrooted phylogenetic tree was constructed from alignments of their domain protein sequences, which resulted in the formation of three distinct clusters: group I, group II, and group III (Figure 3). WRKY proteins of *Arabidopsis* and soybean were present in all clusters. This classification was consistent with results of Rushton et al. [20], who suggested that WRKY domains could be classified into three large groups corresponding to groups I, II, and III of *Arabidopsis*. Notably, *At*WRKY members were more similar to those in the same class in divergent species than they were to other WRKY proteins in the same species. In order to examine the syntenic relationship of the WRKY gene family between the genomes of soybean and *Arabidopsis*, each WRKY gene within the family in *Arabidopsis* was searched in the PGDD (http://chibba.agtec.uga.edu/duplication/) (data no shown). During the course of this analysis we found that synteny was relatively well conserved between soybean and *Arabidopsis* proteins. For example, in subgroup II a (Figure 3), several *Gm*WRKYs (*Glyma06g06530*, *Glyma07g02630*, *Glyma08g23380 Glyma13g44730*, and *Glyma15g00570*) located on different chromosomes are orthologs of a same *At*WRKY gene (*At1g80840*). Additionally, it is worth noting that the structure and phylogenetic tree of the *Gm*WRKY domain clearly indicated that group II proteins could be divided into five distinct subgroups (II a-e).

The phylogenetic classification was found to be consistent with the motif composition among group or subgroup. Differences between groups or subgroups were observed in not only the type of motifs in one WRKY protein, but also in the motif number in one WRKY protein. As displayed schematically in Additional file 2 and Additional file 3, nine types of motifs were detected, including three types of WRKY motifs. The majority of the proteins of subgroups I (73.9%; 17 of 23), II c (93.8%; 30 of 32) and II d (93.3%; 14 of 15), together with those of group III (87.5%; 14 of 16), share a unique WRKY motif, which is shown in red color in Additional file 3. Subgroups II a and II b have the same motif components, suggesting a close phylogenetic relationship. The motif number in each WRKY protein ranged from two to six, and this difference is apparent in groups or subgroups of the WRKY family. For example, all members of group III and the majority of subgroups II e and II d members have two motifs, including a WRKY motif. Interestingly, the relative motif positions in different groups or subgroups also vary significantly. Therefore, motif composition can shed light on phylogenetic relationships of the WRKY family.

Comparison of full-length cDNA sequences with corresponding genomic DNA sequences suggested that the exon number of soybean WRKY genes ranged from two to eight. The results of intron/exon structure identification (Additional file 4) showed that most of the WRKY domain-containing protein genes in different groups or subgroups have very conservative exon numbers. Members of group II d have three exons, and 14 of the 16 genes in group III have three exons. Notably, except for their differences in exon numbers, the relative exon positions in different groups or subgroups also vary significantly. The exon/intron analysis showed clear differences in both exon positions and exon numbers across the different groups or subgroups.

Together, these results indicated that soybean WRKY domains could be classified into three large groups: group I, group II, including group II a-e, and group III. Basic information of all soybean WRKY family members, including conserved heptapeptide, zinc-finger type, domain number, group, coding sequence (CDS) length, and gene length, is provided in Additional file 1.

### Transcriptome atlas and promoter analysis

**Figure 4:**
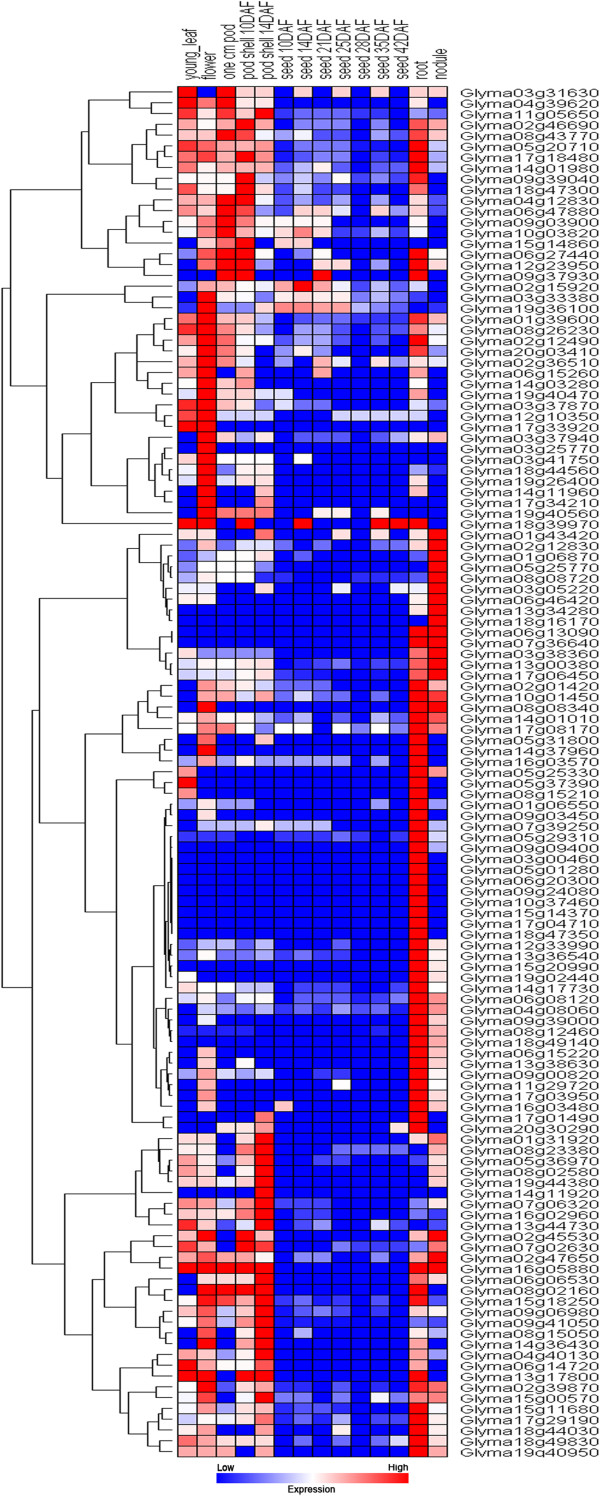
**Expression profiles of 127 soybean WRKY genes.** The hierarchical cluster color code: the largest values are displayed as the reddest (hot), the smallest values are displayed as the bluest (cool), and the intermediate values are a lighter color of either blue or red. Pearson correlation clustering was used to group the developmentally regulated genes. Six genes were excluded from the analysis due to no expression in an organ or a period.

Since the transcriptome sequencing of soybean was completed, the availability of the soybean gene expression atlas facilitates additional studies on the basic biology of soybean [44]. The recently developed RNA-Seq web-based tools, which include gene expression data across multiple tissues and organs, allow for characterization and comparisons of the gene transcriptome atlas in soybean. Consequently, distinct transcript abundance patterns are readily identifiable in the RNA-Seq atlas data set for all 133 *Gm*WRKYs. Similar to other genes that encode transcription factors, many of the GmWRKYs exhibited low transcript abundance levels, as determined by the RNA-Seq atlas analysis. Furthermore, we observed that most of the genes had very broad expression spectra. However, six *Gm*WRKYs, including *Glyma04g05700*, *Glyma04g39650*, *Glyma04g40120*, *Glyma08g01430*, *Glyma01g05050*, and *Glyma14g11440*, lacked expression information, which possibly indicated that these were pseudogenes or were expressed only at specific developmental stages or under special conditions. We observed that accumulation of WRKY gene transcripts was associated with different tissues, and expression patterns differed among each WRKY gene member (Figure 4). In soybean, 33.1% (44 of 133) of the analyzed WRKYs were constitutively expressed in all of the seven tissues tested, which suggested that *Gm*WRKYs play regulatory roles at multiple developmental stages. By contrast, most *Gm*WRKYs showed preferential expression. RNA-Seq atlas data revealed that the majority (92 of 133; 69.2%) of *Gm*WRKYs exhibited transcript abundance profiles with marked peaks in only a single tissue. This result suggests that these WRKY proteins function as tissue-specific regulators and are limited to discrete cells or organs. Approximately 45 of these 133 (33.8%) *Gm*WRKYs showed the highest transcript accumulation level in root tissue, 20 (15.0%) showed the highest transcript accumulation in flower tissue, 13 (9.8%) showed the highest transcript accumulation level in nodule tissue, and surprisingly, only one showed the highest transcript accumulation level in seed tissue. The wide expression of these genes suggests that WRKY genes from soybean are involved in the development of all organs or tissues under normal conditions. In addition to groups of genes that exhibited similar transcript abundance profiles but were relatively phylogenetically distinct, several phylogenetic clades shared the same transcript abundance profile to a large extent. For example, in subgroup II b, most of the *Gm*WRKYs were preferentially expressed in root tissue. Interestingly, in *Arabidopsis*, according to the experimental results of Dong et al. [45], more than half of the members in subgroup II b showed similar preferential expression in the root tissue under normal conditions, which indicated the conserved functional role of subgroup II b in root development between the two species. The expression of members of group I in soybean was detectable in flower tissue, which suggested their conserved roles in flower formation. Members of group I also showed similar expression patterns in nodule and root development. Furthermore, *Gm*WRKYs with high sequence similarity and shared expression profiles represent good candidates for evaluation of gene functions in soybean. The transcriptome atlas indicted that differential expression was extended to all groups or subgroups of the soybean WRKY gene family, which was further verified by the promoter analysis. Because transcription factors bind to corresponding transcription factor binding sites (TFBSs) upstream of genes of interest, profiles of *cis*-acting elements may thus provide useful information related to the regulatory mechanism of gene expression. A computational tool, PlantCARE [46], was adopted to identify putative TFBSs in the 1500-bp DNA sequence upstream of the translation initiation codon of WRKY genes in soybean. Four types of *cis*-acting elements were found to be significantly abundant in the promoter region of *Gm*WRKY genes (Additional file 5). The first type of *cis*-acting element enriched in the promoter region is the light responsive elements, such as G-box [47,48], GAG-motif [49], Box I [50], and Box 4 [51], etc. The G-box element appears to be more abundant in subgroup II a, in which each member contains at least two copies. In addition, the mean number of G-box copies was 3.625 in subgroup II a, which is higher than that in other subgroups or in the whole WRKY gene family. This result indicates that the G-box element seems to be enriched in subgroup II a. All but seven (94.7%; 126 of 133, Group I; 2, Group II b; 2, Group II c; 1, Group II d; 1, and *Glyma14g37960*; 1) have at least one Box 4 element copy. Its mean number of copies (3.744) in the whole WRKY gene family was apparently higher than that of other types of *cis*-acting elements except for TATA-box, CAAT-box, and unnamed-4. This result suggests that the Box 4 element tends to be enriched in the soybean WRKY gene family. As one part of a conserved DNA module involved in light responsiveness, previous studies showed that the Box 4 element is frequently found in promoter regions of different genes from various species [51,52]. However, it is noteworthy that the Box 4 element was found in high frequency in the soybean WRKY gene family, which suggests that the Box 4 element may be important for light-controlled transcriptional activity [53]. Plant hormone responsive elements, such as ABRE [54], P-box [55], as well as the TCA-element [56], constitute the second class. ABRE (51.9%; 69 of 133) appears to be one of the most abundant hormone-related *cis*-acting element in soybean, suggesting that abscisic acid (ABA) regulates the expression of some *Gm*WRKYs, whereas such elements were rarely detected in Group II d (20.0%; 3 of 15). By contrast, the salicylic acid responsive TCA-element was frequently found in groups or subgroups. These observations suggest that *Gm*WRKYs in different groups are likely to be significantly regulated by different types of hormones. The third most abundant *cis*-acting element class consisted of elements that respond to external environmental stresses. We observed that most of the *Gm*WRKYs examined appeared to contain MBS (72.2%; 96 of 133) [57], heat shock element (HSE) (77.4%; 103 of 133) [58], and TC-rich repeat elements (71.4%; 95 of 133) [59]. MBS is an element involved in drought induction, and HSE is also enriched in the promoter. With a few exceptions, *Gm*WRKYs contain more than two copies of this element. Circadian elements, which are involved in circadian control [60], is the fourth type of *cis*-acting element that was abundantly found in the promoter regions of soybean WRKY genes. PlantCARE identified 98 (73.7%; 98 of 133) *Gm*WRKY genes containing circadian elements, which may be responsible for its distinct diurnal expression pattern. The presence of a diversity of *cis*-acting elements in the upstream regions of *Gm*WRKYs indicates that *Gm*WRKYs may function in a relatively wide range of activities.

The above results indicated that the 133 WRKY genes in soybean display differential expression, either in their transcript abundance or in their expression patterns under normal growth conditions in different groups or subgroups.

### Detection of positive selection and functional divergence analysis (FDA)

Site models and branch-site models in PAML [61] were used to detect positive selection in the WRKY gene family of soybean. Substitution rate ratios of non-synonymous (dN or Ka) versus synonymous (dS or Ks) mutations (dN/dS or ω) were calculated. A Ka/Ks ratio of 1 indicates genes that are subject to neutral selection, <1 indicates genes subject to negative selection, and >1 indicates genes subject to positive selection [62]. Additional file 6 lists parameter estimates and log-likelihood values for each site model. Two pairs of models (M0/M3 and M7/M8) were selected and compared. The site-homogeneous model, M0 (one-ratio), assumes one ω for all sites, whereas M3 (discrete) assumes a general discrete distribution. Two other models used were M7 (beta), which assumes a beta distribution of ω that is limited to the range (0, 1), and M8 (beta & ω), which adds an extra site class with ω estimated [63]. In addition, to test for variable omega ratios among lineages, we conducted the likelihood ratio test (LRT) to compare the two extreme models. The log likelihood values under the one-ratio model and the discrete model were determined to be −8608.409 and −8272.457, respectively. Twice the log likelihood difference value, 2ΔlnL = 335.95, was found to be strongly statistically significant (p < 0.01), thus revealing a heterogeneous selective pressure among lineages. Moreover, the log likelihood value under the beta model and the beta & ω were −8253.022 and −10197.202, respectively. Twice the log likelihood difference, 2ΔlnL = 1944.18, was also strongly statistically significant (p < 0.01). The comparison of M3 versus M0 revealed that none of the codon sites appeared to be under the influence of positive selection (ω > 1). By contrast, comparing the M7 model to the M8 model indicated that ~0.001% of codons fell within an estimated ω value of 2.638, suggesting positive selection. We also used Bayes empirical Bayes (BEB) estimation methods in model M8 [64] to identify sites under positive selection. We found only one positive selection site at the 0.05 significance level, and three sites at the 0.01 significance level. Together, these results indicate that no strong positive selection sites could be detected under the site model in the soybean WRKY gene family.

Branch-site models allow ω to vary both among sites in the protein and across branches on the tree, and aim to detect positive selection affecting a few sites along particular lineages [64]. The branches being tested for positive selection are referred to as the foreground branches, and all other branches on the tree are referred to as background branches. The BEB method was implemented to calculate posterior probabilities (Qks) for site classes if the LRT suggested the presence of codons under positive selection on the foreground branch [65]. In our study, group I, group II a-e, and group III were selected as foreground branches, respectively, while the other groups were selected as the background branches. It is notable that no positive selection sites were observed in groups II a, II b, or II d. In contrast, positive selection sites detected by the branch-site model (Table 1) were distributed in groups II e and III at the 0.01 significance level. This result demonstrated that the groups have different evolutionary rates. Group II e and group III appeared to be confronted with strong positive Darwinian selection, as many highly significant positive sites were present, whereas evolution in the other groups appeared to be more conservative.

**Table 1:** Parameters estimation and likelihood ratio tests for the branch-site models

**Branch-site model**					
**Foreground branches**	**Estimates of parameter**				**Positive selection sites (BEB) **^**4**^
	**Site class**^**1**^**0**	**Site class 1**	**Site class 2a**	**Site class 2b**	
Group 1	P_0_ = 0.75959	P_1_ = 0.05958	P_2a_ = 0.16768	P_2b_ = 0.01315	261G*,262S*,282H*,
	ω_0(b)_^2^ = 0.05978	ω_1(b)_ = 1.00000	ω_2a(b)_ = 0.05978	ω_2b(b)_ = 1.00000	288D*, 292 M*
	ω_0(f)_^3^ = 0.05978	ω_1(f)_ = 1.00000	ω_2a(f)_ = 232.95169	ω_2b(f)_ = 232.95169	
Group 2a	P_0_ = 0.00000	P_1_ = 0.00000	P_2a_ = 0.92727	P_2b_ = 0.07273	None
	ω_0(b_) = 0.06077	ω_1(b)_ = 1.00000	ω_2a(b)_ = 0.06077	ω_2b(b)_ = 1.00000	
	ω_0(f)_ = 0.06077	ω_1(f)_ = 1.00000	ω_2a(f)_ = 999.00000	ω_2b(f)_ = 999.00000	
Group 2b	P_0_ = 0.00001	P_1_ = 0.00000	P_2a_ = 0.92726	P_2b_ = 0.07273	None
	ω_0(b)_ = 0.06039	ω_1(b)_ = 1.00000	ω_2a(b)_ = 0.06039	ω_2b(b_) = 1.00000	
	ω_0(f)_ = 0.06039	ω_1(f)_ = 1.00000	ω_2a(f)_ = 1.00000	ω_2b(f)_ = 1.00000	
Group 2c	P_0_ = 0.80507	P_1_ = 0.06312	P_2a_ = 0.12223	P_2b_ = 0.00958	261G**,275R**
	ω_0(b_) = 0.06042	ω_1(b)_ = 1.00000	ω_2a(b)_ = 0.06042	ω_2b(b)_ = 1.00000	
	ω_0(f)_ = 0.06042	ω_1(f)_ = 1.00000	ω_2a(f)_ = 167.23585	ω_2b(f)_ = 167.23585	
Group 2d	P_0_ = 0.00000	P_1_ = 0.00000	P_2a_ = 0.92727	P_2b_ = 0.07273	None
	ω_0(b)_ = 0.06118	ω_1(b)_ = 1.00000	ω_2a(b)_ = 0.06118	ω_2b(b)_ = 1.00000	
	ω_0(f)_ = 0.06118	ω_1(f)_ = 1.00000	ω_2a(f)_ = 981.94932	ω_2b(f)_ = 981.94932	
Group 2e	P_0_ = 0.76730	P_1_ = 0.06008	P_2a_ = 0.16008	P_2b_ = 0.01253	248E**, 249Y**, 286A*,
	ω_0(b_) = 0.06061	ω_1(b)_ = 1.00000	ω_2a(b)_ = 0.06061	ω_2b(b)_ = 1.00000	288D**, 298E**, 299G**
	ω_0(f)_ = 0.06061	ω_1(f)_ = 1.00000	ω_2a(f)_ = 999.00000	ω_2b(f)_ = 999.00000	
Group 3	P_0_ = 0.63465	P_1_ = 0.04978	P_2a_ = 0.29262	P_2b_ = 0.02295	258P*, 260 K**, 263P**, 275R*,
	ω_0(b)_ = 0.06176	ω_1(b)_ = 1.00000	ω_2a(b)_ = 0.06176	ω_2b(b)_ = 1.00000	293 L*, 294I**, 298E**, 303H**
	ω_0(f)_ = 0.06176	ω_1(f)_ = 1.00000	ω_2a(f)_ = 999.00000	ω_2b(f)_ = 999.00000	

Note: * p < 0.05 and ** p < 0.01 (*x*^2^ test).

1 The sites in the sequence evolve according to the same process, the transition probability matrix is calculated only once for all sites for each branch.

2 Background ω.

3 Foreground ω.

4 The number of amino acid sites estimated to have undergone positive selection; BEB: Bayes Empirical Bayes.

Type I functional divergence (shifted evolutionary rate) and Type II functional divergence (altered amino acid physicochemical property) between gene clusters of the WRKY gene family were estimated by posterior analysis using the program DIVERGE v2.0 [66,67]. Because these methods are not sensitive to saturation of synonymous sites, they have been extensively applied in research of various gene families [68-70]. The estimation was based on the WRKY protein neighbor-joining tree, in which seven major clades were clearly present. Pairwise comparisons of paralogous WRKY genes from group I, group II a-e, and group III were carried out, and the rate of amino acid evolution at each sequence position was estimated. Our results (Additional file 7) indicated that with nine exceptions (group pairs II d/II e, II d/III, II d/II a, II d/I, II e/III, III/II a, and II b/I), the coefficients of Type-I functional divergence (θ_ML_) between WRKY groups were moderately statistically significant (p < 0.05), with θ_ML_ values ranging from 0.201 to 0.395, or strongly statistically significant (p < 0.01) with θ _ML_ values ranging from 0.311 to 0.618. These results indicated significant site-specific altered selective constraints on some members of the WRKY family, leading to subgroup-specific functional evolution after diversification. Additionally, Type-I functional divergence was not evident in the comparison of group II d with the other four groups, which suggests that group II d may be the most conservative clade. Type-II functional divergence was evident in all groups or subgroups (Additional file 8), with θ-II values ranging from 0.033 to 0.288 (p < 0.05), indicating a radical shift of amino acid properties. These results suggest that the relative importance of Type-I and Type-II functional divergence might be associated with specific functional classes of the WRKY gene family in soybean.

Furthermore, some critical amino acid residues responsible for functional divergence were predicted with suitable cut-off values derived from the Qk of each comparison. In order to reduce false positives, Qk > 0.8 was used as the cutoff to identify Type-I and Type-II functional divergence-related residues in all comparisons between the seven WRKY groups or subgroups. Results showed distinct differences in the number and distribution of predicted sites for functional divergence within each pairwise comparison. However, some critical amino acid sites still showed evidence of both Type-I and Type-II functional divergence in corresponding pairs. For example, five critical residues were predicted for the subgroup II e/II c (248E, 258P, 264Y, 275R, 295 V) and II e/II b (248E, 258P, 275R, 276G, 298E) pairwise comparisons, whereas three critical amino acids sites were predicted for the subgroup II e/I (248E, 264Y, 275R) and III/II b (264Y, 295 V, 298E) pairwise comparisons, respectively. Similar cases were found in other subgroup pairwise comparisons. Shifted evolutionary rates and altered amino acid physicochemical properties co-occurred at the same amino acid sites, revealing that these sites have played important roles in functional divergence during the process of evolution.

### Expansion pattern of the WRKY family in soybean

Gene duplication events are important for gene family evolution, because duplicated genes provide the raw materials for the generation of new genes, which in turn facilitate the generation of new functions. Three principal evolutionary patterns were attributed to gene duplications: segmental duplication, tandem duplication, and transposition events such as retroposition and replicative transposition [71]. Among these, segmental duplication occurs most frequently in plants because most plants are diploidized polyploids and retain numerous duplicated chromosomal blocks within their genomes [72]. Previous studies reported several rounds of whole-genome duplication (WGD) in both the *Arabidopsis* and rice genomes [73,74]. The occurrence of large-scale gene duplication events was also demonstrated in soybean [43]. For this analysis, we focused on the tandem and segmental duplication modes. Tandem duplications were characterized as multiple members of one family occurring within the same intergenic region or in neighboring intergenic regions, where genes were clustered together with a maximum of 10 extra genes between them [40]. We searched for contiguous WRKY genes in both the sharing region and neighboring regions. Eighteen of the 133 genes (13.5%) in this family were found to be located as tandem repeats in soybean (Figure 1), indicating that tandem duplications contributed to the expansion of this family. We also tested the hypothesis that segmental duplication events played a large role in the evolution of the WRKY gene family in soybean. For each WRKY gene, we tallied the number of flanking protein-coding genes with a best non-self match to a protein-coding gene neighboring its paralog. According to our results, 76.7% (102 of 133) of genes showed high conservation, indicating that these WRKY genes were formed through segmental duplication in soybean (Table 2). Intriguingly, comparison of the 102 segmental duplicated genes in our study to the results of Du et al. [75] suggested that 91 (89.2%; 91 of 102) WRKY genes originated from WGDs, and the duplication status of the remaining 11 (10.7%; 11 of 102) WRKY genes, including *Glyma01g05050*, *Glyma01g43420*, *Glyma02g15920*, *Glyma02g45530*, *Glyma03g00460*, *Glyma03g31630*, *Glyma08g15210*, *Glyma10g03820*, *Glyma13g38630*, *Glyma18g16170*, and *Glyma18g44030*, was singleton, which indicated that these segmental duplication genes may be derived from independent duplication events. These results indicated that most of the WRKY genes in soybean were retained after WGDs. Edger et al. [76] stated that dosage-sensitive genes, including transcription factors, were preferentially retained following WGDs, which is compatible with the present study. We did not find evidence that other pairs of paralogous genes in soybean originated from segmental duplication. These results indicate that the soybean WRKY family arose mainly through segmental duplications.

**Table 2:** Estimates of the dates for the segmental duplication events of WRKY family in soybean

**Segment pairs**	**Number of anchors**	**KS (mean ± s.d.)**	**Estimated time (mya)**
Glyma01g05050 & Glyma18g16170	4	0.60 ± 0.20	49
Glyma01g06550 & Glyma02g12490	3	0.17 ± 0.06	14
Glyma01g06870 & Glyma02g12830	4	0.16 ± 0.08	13
Glyma01g31920 & Glyma03g05220	5	0.19 ± 0.06	16
Glyma01g31920 & Glyma18g44030	3	0.71 ± 0.22	58
Glyma01g39600 & Glyma11g05650	17	0.17 ± 0.07	14
Glyma01g43420 & Glyma05g36970	5	0.70 ± 0.19	57
Glyma01g43420 & Glyma08g02580	4	0.68 ± 0.17	56
Glyma02g01420 & Glyma03g37940	8	0.67 ± 0.11	55
Glyma02g01420 & Glyma10g01450	19	0.20 ± 0.11	16
Glyma02g01420 & Glyma19g40560	7	0.72 ± 0.17	59
Glyma02g15920 & Glyma03g31630	6	0.60 ± 0.16	49
Glyma02g15920 & Glyma10g03820	11	0.15 ± 0.11	12
Glyma02g36510 & Glyma17g08170	18	0.13 ± 0.05	11
Glyma02g45530 & Glyma14g03280	6	0.12 ± 0.05	10
Glyma02g46690 & Glyma08g43770	8	0.61 ± 0.19	50
Glyma02g46690 & Glyma14g01980	16	0.13 ± 0.07	11
Glyma02g47650 & Glyma14g01010	21	0.14 ± 0.08	11
Glyma03g00460 & Glyma09g41050	5	0.51 ± 0.12	42
Glyma03g31630 & Glyma10g03820	6	0.55 ± 0.10	45
Glyma03g33380 & Glyma19g36100	21	0.19 ± 0.17	16
Glyma03g37870 & Glyma19g40470	18	0.15 ± 0.07	12
Glyma03g37940 & Glyma10g01450	9	0.73 ± 0.16	60
Glyma03g37940 & Glyma19g40560	17	0.15 ± 0.07	12
Glyma03g38360 & Glyma19g40950	11	0.16 ± 0.09	13
Glyma03g41750 & Glyma07g06320	5	0.66 ± 0.23	54
Glyma03g41750 & Glyma16g02960	3	0.62 ± 0.26	51
Glyma03g41750 & Glyma19g44380	19	0.18 ± 0.13	15
Glyma04g08060 & Glyma06g08120	14	0.18 ± 0.12	15
Glyma04g39620 & Glyma06g15260	14	0.23 ± 0.19	19
Glyma04g39650 & Glyma05g31800	4	0.72 ± 0.19	59
Glyma04g39650 & Glyma06g15220	15	0.22 ± 0.19	18
Glyma04g39650 & Glyma08g15050	4	0.66 ± 0.19	54
Glyma04g40130 & Glyma06g14720	18	0.25 ± 0.25	20
Glyma05g01280 & Glyma06g20300	6	0.57 ± 0.21	47
Glyma05g25330 & Glyma08g08340	12	0.21 ± 0.19	17
Glyma05g25770 & Glyma08g08720	14	0.20 ± 0.15	16
Glyma05g29310 & Glyma08g12460	18	0.16 ± 0.07	13
Glyma05g31800 & Glyma06g15220	4	0.63 ± 0.22	52
Glyma05g31800 & Glyma08g15050	19	0.14 ± 0.08	11
Glyma05g36970 & Glyma08g02580	17	0.22 ± 0.15	18
Glyma05g37390 & Glyma08g02160	17	0.14 ± 0.08	11
Glyma06g15220 & Glyma08g15050	5	0.68 ± 0.23	56
Glyma06g15260 & Glyma08g15210	3	0.71 ± 0.21	58
Glyma06g46420 & Glyma12g10350	9	0.23 ± 0.19	19
Glyma06g46420 & Glyma13g38630	6	0.72 ± 0.11	59
Glyma07g02630 & Glyma08g23380	15	0.22 ± 0.18	18
Glyma07g02630 & Glyma13g44730	6	0.56 ± 0.18	46
Glyma07g02630 & Glyma15g00570	6	0.54 ± 0.15	44
Glyma07g06320 & Glyma16g02960	12	0.16 ± 0.07	13
Glyma07g06320 & Glyma19g44380	5	0.61 ± 0.17	50
Glyma07g36640 & Glyma15g14860	5	0.59 ± 0.21	48
Glyma07g36640 & Glyma17g03950	13	0.23 ± 0.23	19
Glyma07g36640 & Glyma09g03900	5	0.73 ± 0.18	60
Glyma07g39250 & Glyma09g00820	5	0.63 ± 0.15	52
Glyma07g39250 & Glyma15g11680	7	0.65 ± 0.18	53
Glyma07g39250 & Glyma17g01490	23	0.17 ± 0.11	14
Glyma08g23380 & Glyma13g44730	4	0.49 ± 0.18	40
Glyma08g23380 & Glyma15g00570	4	0.52 ± 0.17	43
Glyma08g26230 & Glyma18g49830	8	0.21 ± 0.08	17
Glyma08g43770 & Glyma14g01980	7	0.56 ± 0.13	46
Glyma09g00820 & Glyma15g11680	15	0.20 ± 0.14	16
Glyma09g00820 & Glyma17g01490	5	0.67 ± 0.21	55
Glyma09g03450 & Glyma15g14370	14	0.20 ± 0.15	16
Glyma09g03900 & Glyma15g14860	12	0.25 ± 0.21	20
Glyma09g03900 & Glyma17g03950	3	0.59 ± 0.17	48
Glyma09g06980 & Glyma13g00380	5	0.69 ± 0.18	57
Glyma09g06980 & Glyma15g18250	7	0.22 ± 0.21	18
Glyma09g06980 & Glyma17g06450	5	0.56 ± 0.11	46
Glyma09g39000 & Glyma18g47350	11	0.19 ± 0.10	16
Glyma09g39040 & Glyma18g47300	13	0.18 ± 0.10	15
Glyma09g41050 & Glyma18g44560	12	0.16 ± 0.06	13
Glyma10g01450 & Glyma19g40560	6	0.70 ± 0.17	57
Glyma10g37460 & Glyma20g30290	11	0.13 ± 0.05	11
Glyma11g05650 & Glyma17g18480	3	0.71 ± 0.23	58
Glyma12g10350 & Glyma13g38630	4	0.73 ± 0.10	60
Glyma12g33990 & Glyma13g36540	12	0.23 ± 0.20	19
Glyma13g00380 & Glyma17g06450	17	0.19 ± 0.13	16
Glyma13g17800 & Glyma17g04710	17	0.20 ± 0.17	16
Glyma13g44730 & Glyma15g00570	13	0.12 ± 0.06	10
Glyma14g11440 & Glyma17g34210	6	0.32 ± 0.19	26
Glyma14g11920 & Glyma17g33920	5	0.17 ± 0.05	14
Glyma15g11680 & Glyma17g01490	7	0.67 ± 0.20	55
Glyma15g14860 & Glyma17g03950	5	0.58 ± 0.24	48
Glyma15g18250 & Glyma17g06450	4	0.68 ± 0.16	56
Glyma16g02960 & Glyma19g44380	3	0.58 ± 0.17	48

Abbreviation: mya, million years ago.

We also used Ks, as the proxy for time, and the conserved flanking protein-coding genes to estimate the dates of the segmental duplication events. The mean Ks values and the estimated dates for all segmental duplication events corresponding to WRKY genes are listed in Table 2. The segmental duplicated events in soybean appear to have occurred recently, and focus on two periods, 10–20 mya and 40–60 mya, which is consistent with the ages of the genome duplication events [43]. Taken together, our analyses suggested that segmental duplication is the main mechanism for expansion of this WRKY gene family, accompanied by tandem duplications.

## Discussion

### Identification, classification, and phylogenetic analysis of the soybean WRKY gene family

The genome sequence and transcriptome profiles of soybean provide a large amount of useful data to explore functional diversity from multiple perspectives. In this study, we identified 133 WRKY members in the soybean genome. A phylogenetic tree including 133 distinct protein sequences clearly demonstrated that these genes could be divided into three groups. This classification was further supported by the results of motifs and exon/intron analyses. The topology of our phylogenetic tree constructed from WRKY genes of two species (soybean and *Arabidopsis*) is largely consistent with that derived from *Arabidopsis* alone. All of the evidence obtained suggested that the classification was reasonable and reliable.

WRKY transcription factors have their evolutionary origin in ancient eukaryotes, whose genomes contain a single WRKY gene with two WRKY domains. The presence of a group I WRKY protein in these ancient organisms suggests that group I WRKY genes represent the ancestral form, with other groups arising later through losses and gains of WRKY domains [22]. In the present study, phylogenetic analysis based on the relationship of different groups, indicated that domain gain and loss has indeed been a driving force in the expansion of the WRKY gene family. For example, subgroups II a, II b, and II c were phylogenetically closer to the C-terminal WRKY domain of group I.

*Glyma14g37960* and *Glyma18g39970* were not assigned to any groups or subgroups. *Glyma14g37960* has one WRKY domain; however, it is phylogenetically closer to group I N. Thus, *Glyma14g37960* may have arisen from a two-domain WRKY protein that lost one of its WRKY domains during evolution, whereas in *Glyma18g39970*, a mutation in the sequence outside of the WRKY domain may have occurred before or after the domain loss.

### Transcriptome atlas, positive selection, and FDA of soybean WRKY proteins

The transcriptome atlas revealed differential expression of the WRKY gene family under normal growth conditions. Furthermore, results of the promoter analysis were compatible with differential expression patterns. The elements were distributed across three main functional categories, including biotic and abiotic stresses and developmental processes. Surprisingly, Skn-1 motif elements, which are required for high levels of endosperm expression in cooperative interaction with other motifs (AACA, GCN4, ACGT) [77], were found to be abundant in all groups or subgroups. This result appears to contradict with the expression analysis, in which only one gene showed the highest transcript accumulation level in seed tissue. Since the function of *cis*-acting elements is to regulate gene expression, we speculated that the reason for this phenomenon might be due to the deficiency of Skn-1 motif element partners, AACA and ACGT elements, which were rarely detected in our study. On the other hand, according to the transcriptome atlas of the soybean WRKY gene family, the majority of *Gm*WRKYs showed relatively reduced expression in seed development compared to other organs, which suggests that the expression of genes can be significantly affected when the Skn-1 motif lacks its partners. To further investigate the reason for this differential expression, we performed a positive selection analysis and a functional divergence analysis.

We used both site models and branch-site models to detect positive selection. The results of site models indicated that one category of ω did not fit the data well to describe the variability in selection pressures across amino acid sites. Therefore, the branch-site models, which allowed ω ratios to vary among sites and lineages simultaneously, appeared to be most suitable for describing evolutionary processes of the WRKY gene family. The branch-site analysis revealed that several sites were under positive selection. Along the group II e clade, the following sites were identified to be under positive selection: 248E, 249Y, 286A, 288D, 298E, and 299G. Similar results were found in the group I, III, and II c clades.

**Figure 5:**
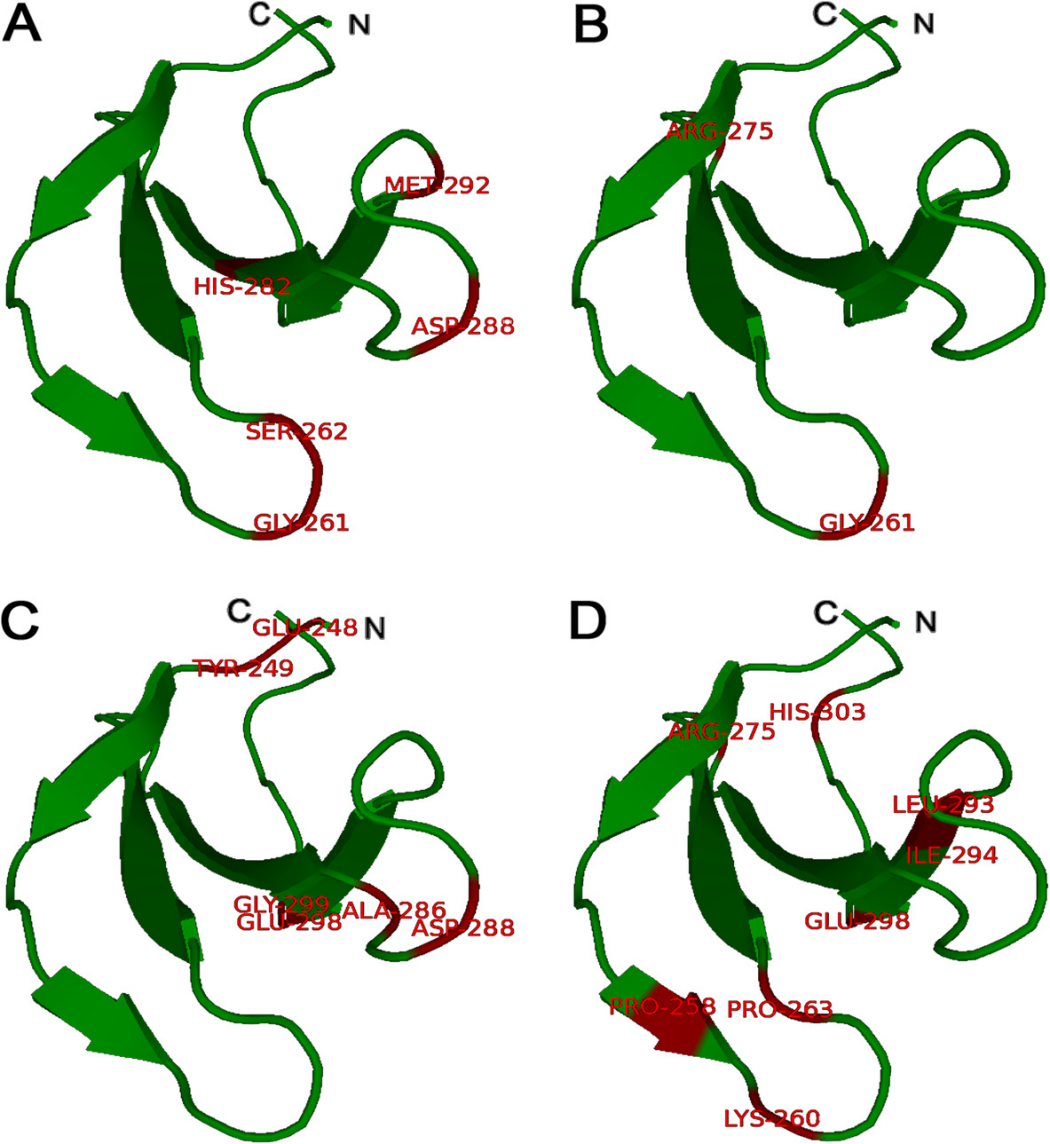
**Model building of the 3D structure of the soybean WRKY protein (*Glyma13g00380*) based on similarity to the*At*WRKY4-C domain (Protein Data Bank (PDB) code: 2lexA).** The ensemble of the selected structures in stereo view **(A)**, **(B)**, **(C)**, and **(D)** positive selection sites detected by the branch-site model presented in group I, group II c, group II e, and group III, respectively. The sites with red color indicate amino acid residues under statistically significant (p < 0.05) positive selection, as calculated by Bayes Empirical Bayes estimation methods. The presented region is Asp247–Pro306, excluding the N-terminal region. The figure was produced using the Swiss-model and pyMOL programs.

Figure 5 shows the locations of amino acid sites detected by PAML 4 in the 3D structure. Interestingly, with the exception of four amino acid sites (position 258, 282, 293, and 294), sites in different groups or subgroups were all located in the loop regions. Duan et al. [78] suggested that the DNA-binding ability of *At*WRKY was mediated through the beta-hairpin regions between β2 and β3, and similar results were reported by Maeo et al. [79]. These results confirmed the theory proposed by Church et al. [80] for non-helical DNA binding. Furthermore, previous work on DNA binding of the WRKY family revealed that the conserved WRKYGQK region was important for DNA binding [79]. According to our results, the amino acid residues of bridging loops between β-strand regions may have been adapted for new functional roles during the process of evolution.

Moreover, we further compared the number of WRKY genes in different groups or subgroups among *Arabidopsis*, rice, and soybean (Table 3). We observed that the number of members in different groups or subgroups was approximately doubled in soybean than in corresponding groups or subgroups in *Arabidopsis* and rice, which can be attributed to the more recent two genome duplication events in the soybean genome [43]. The key difference is that the number of group III members in soybean is roughly the same as that in *Arabidopsis*, but half of that in rice. This result may indirectly reflect the fact that group III in the dicotyledons may be confronted with strong positive Darwinian selection, whereas the evolution of this subgroup may be more conservative in the monocotyledons.

**Table 3:** **Number of WRKY genes in *Arabidopsis, *rice, and soybean**

	**Group1**	**Group2a**	**Group2b**	**Group2c**	**Group2d**	**Group2e**	**Group3**
*At*WRKY	13	4	7	18	7	9	14
*Os*WRKY	15	4	8	15	7	11	36
*Gm*WRKY※	23	8	19	32	15	18	16

Note: ※ the WRKY proteins of soybean (*Glycine max*).

Functional innovations include subfunctionalization [81], neofunctionalization [82], and subneofunctionalization [83]. Gene duplication may result in altered functional constraints between the gene clusters of a gene family. The results of the functional divergence analysis suggested that WRKY genes should be significantly functionally divergent from each other, especially with respect to the four amino acid residues (248E, 275R, 288D and 298E) identified by both PAML 4 and DIVERGE 2.0 analyses, which were inferred to have played important roles during evolution. On the other hand, functional divergence might reflect the existence of long-term selective pressures.

### The soybean WRKY gene family arose mainly through segmental duplication

The dramatic variation we observe in gene family size and distribution may have resulted owing to many processes, including tandem duplication with high rates of birth and death and gene duplication resulting from larger scale genome events such as polyploidy or duplications of chromosomal regions (here referred to as “segmental duplications”).

The current investigation revealed the duplication pattern of the soybean WRKY gene family. One hundred two genes were found to evolve from segmental duplication, suggesting that segmental duplication likely played a pivotal role in WRKY gene expansion in the soybean genome. The genome sequencing results revealed two genome duplication events in soybean, aging ~13 mya and ~57 mya [43], which is consistent with results of the present study. We inferred that the expansion of the WRKY gene family occurred along with genome duplication events, and that these genes were retained during evolution. The structural similarity and variation between genes located on the same chromosome and the phylogenetic analysis might help to explain the order of duplication events of the soybean WRKY genes on the same chromosome. For example, *Glyma02g01420*, *Glyma02g12830*, and *Glyma02g15920*, which are located in different duplication blocks of the same chromosome, all have two introns flanked by three exons. However, phylogenetic analysis showed that *Glyma02g01420* was more similar to *Glyma10g01450*, and *Glyma02g15920* was more similar to *Glyma10g03820*, whereas *Glyma02g12830*, which is located relatively close to *Glyma02g15920*, had no duplicate genes on chromosome 10. It is possible that the duplication of the same ancestral gene on chromosome 2 resulted in *Glyma02g12830* and the ancestor of *Glyma10g01450* and *Glyma10g03820*, which then evolved independently. The intron and exon sequences of the ancestor gene might have elongated or shorten because of various reasons after it split into *Glyma02g01420* and *Glyma02g15920*. Through segmental duplication, the two chromosome segments, one contained in *Glyma02g12830* and the other contained in *Glyma02g01420* and *Glyma02g15920*, were independently copied to different parts of chromosome 10. During subsequent evolution, the counterpart of *Glyma02g12830* was lost and structures for the counterparts of *Glyma02g01420* and *Glyma02g15920* changed by deletion or insertion of other fragments or partial sequence repeat variations. Moreover, we noticed that both the *Arabidopsis* and rice genomes underwent recent duplication events, which also resulted in large-scale expansion of the WRKY gene family in their genomes. Therefore, we also examined the duplicated pattern of WRKY genes in these model species.

The complete sequencing of the *Arabidopsis* genome revealed numerous large-scale segmental duplications [84]. Previous studies concluded that at least two rounds of duplications probably occurred in the *Arabidopsis* genome, with many losses and rearrangements leaving a mosaic of “segmental duplications” or “duplication blocks” [74,84]. Most duplication blocks appear to have originated from one round of polyploidy, as estimated by using various methods, that occurred 20–40 mya, before the evolution of the genus *Brassica* but after the separation of Brassicaceae from other closely related eudicot families [74]. Results of the present study showed that no apparent tandem duplication events, and rare segmental duplication events (six pairs), exist in the *Arabidopsis* WRKY gene family. Furthermore, the estimated time of the six pairs of segmental duplicated genes focus on the period of 24–27 mya (Additional file 9). Cannon at el. [72] found nine distinct pairs of duplicated segments and no tandem duplication events in the *Arabidopsis* WRKY family, which is compatible with our study. Comparison of six pairs of segmental duplicated genes in our study with the results of Blanc et al. [85] suggested that only one pair of genes (*At1g13960* and *At2g033400*) originated from polyploidy in *Arabidopsis*. Consequently, we speculated that the other five segmental duplicated genes might have derived from independent segmental duplication events. The long period of time over which genome evolution has occurred has provided many opportunities for functional divergence in the genes that arose from duplications. Our results did not reveal evidence that other pairs of WRKY genes in *Arabidopsis* originated from duplicated blocks. Therefore, most of the *Arabidopsis* WRKY genes may have lost their paralogous genes after genome duplication [74].

With respect to rice, the expansion patterns of WRKY gene family have been clearly demonstrated. Ramamoorthy et al. [40] predicted 103 genes encoding WRKY transcription factors in rice, and the majority of rice WRKY genes (77.7%; 80 of 103) were detected on duplicated blocks. Of the WRKY genes, 45.6% (47 of 103) of WRKY genes were found to have corresponding coordinates generated by segmental duplications. Furthermore, 35.0% (36 of 103) of the WRKY genes were clustered together with a maximum of 10 extra genes between them, and were regarded as tandemly duplicated genes. The results above were confirmed by Jiang et al. [86]. That is, that both tandem and segmental duplication significantly contributed to the expansion of the WRKY gene family in rice.

All of the evidence suggests that the evolutionary patterns of the WRKY gene family differ between soybean, rice, and *Arabidopsis*. Species-specific expansion played an important role in the evolution of this family in plants. Segmental duplication appears to be the dominant mechanism for the generation of duplicated genes in soybean, whereas segmental duplication and tandem duplication may play similar roles in the expansion of the rice WRKY gene family. Moreover, although *Arabidopsis* may have a tetraploid ancestor, the majority of its duplicated genes appear to have been lost throughout evolutionary processes.

## Conclusion

Previous studies have demonstrated that members of the WRKY gene family play important roles in the regulation of several plant developmental processes and in responses to various biotic and abiotic stresses. Results of the present study indicate that segmental duplication has likely been the dominant mechanism of gene amplification during the expansion of the WRKY family in soybean. Furthermore, positive selection could be the main driving forces for the functional divergence of duplicated genes, which may have played a critical role in the responses of plants to various stresses throughout their evolutionary history. The results of this study will not only further our understanding of the evolutionary processes of soybean WRKY genes, but will also help to enhance functional genomics studies of WRKY transcription factors in an important model system.

## Methods

### Sequence collection

Seventy WRKY protein sequences downloaded from AtTFDB (http://arabidopsis.med.ohio-state.edu/AtTFDB/) were used to BLAST against the soybean genome database, Phytozome v8.0 (http://www.phytozome.net/soybean), using the BLASTP program. Sequences were selected as candidate proteins if their E value was ≤ 1e-10. For each query sequence, information of the location on chromosomes, genomic sequences, full coding sequences (CDS), and protein sequences were collected from Phytozome, and redundant genes were removed manually. The Simple Modular Architecture Research Tool (SMART; http://smart.embl-heidelberg.de/) was used to confirm each predicted WRKY member.

### Phylogenetic tree construction and sequence analysis

The SMART program was used to extract the protein sequences of the WRKY domain for each protein. Multiple sequence alignment of domain sequences of 133 WRKY family proteins from soybean and 70 protein sequences from *Arabidopsis* was performed using the Clustal X 1.83 program with default parameters, and a phylogenetic tree was generated and viewed using MEGA Version 5.0. Exon and intron organizations of soybean WRKY genes were determined by comparing predicted CDS with their corresponding genomic sequences using GSDS (http://gsds.cbi.pku.edu.cn/) software. Motifs of paralogous WRKY proteins were identified statistically using MEME with default settings, except that the maximum number of motifs to find was set at 10.

### RNA-Seq atlas and promoter analysis

RNA-Seq data were introduced to further analyze the expression of *Gm*WRKY genes. Data was normalized using a variation of the reads/Kb/Million method, and Z-score analysis was obtained from SoyBase (http://soybase.org/soyseq/) [44,87]. A heat map was generated using the GenePattern program (http://www.broadinstitute.org/cancer/software/genepattern/index.html). The *cis*-acting elements that regulate gene expression are distributed in 300–3000 bp upstream of the coding region, also take into consideration of sequence restriction in PlantCARE (http://bioinformatics.psb.ugent.be/webtools/plantcare/html/) and the methods described by Liu et al. [68], therefore, 1500 bp upstream of the coding region were selected as promoter sequence and were downloaded from Phytozome (http://www.phytozome.net) and Soybean Functional Genomics Database (bioinformatics.cau.edu.cn). Then these sequences were submitted to PlantCARE for *in silico* analysis.

### Positive selection and functional divergence

A maximum likelihood method in PAML was applied to test the hypothesis of positive selection in the WRKY gene family [63] under the site model and branch-site model. In the site model, two pairs of models were contrasted to test the selective pressures at codon sites. First, models M0 (one ratio) and M3 (discrete) were compared, using a test for heterogeneity between codon sites in the dN/dS ratio value, ω. The second comparison was M7 (beta) versus M8 (beta + ω > 1). Meanwhile, we introduced the likelihood ratio test (LRT) to compare the two extreme models. When the LRT suggested positive selection, the Bayes empirical Bayes (BEB) method was used to calculate the posterior probabilities that each codon was from the site class of positive selection under models M3 and M8.

The branch-site model assumes that the ω ratio varies between codon sites and that there are four site classes in the sequence. The first class of sites is highly conserved in all lineages with a small ω ratio, ω_0_. The second class includes neutral or weakly constrained sites for which ω = ω_1_, where ω_1_ is near or smaller than 1. In the third and fourth classes, the background lineages show ω_0_ or ω_1_, but foreground branches have ω_2_, which may be greater than 1. When constructing the LRTs, the null hypothesis fixes ω_2_ = 1, allowing sites evolving under negative selection in the background lineages to be released from constraint and to evolve neutrally on the foreground lineage; the alternative hypothesis constrains ω_2_ ≥ 1 [64]. Posterior probabilities (Qks) were calculated using the BEB method [65].

The software DIVERGE was used to reveal the functional divergence between members of the WRKY protein family. The coefficients of Type-I and Type-II functional divergence (θ-I and θ-II, respectively) between any two clusters of interest were calculated. A θ-I or θ-II significantly > 0 indicates site-specific altered selective constraints or a radical shift of amino acid physicochemical properties after gene duplication and/or speciation [66]. Moreover, Qk was used to predict critical amino acid residues that were responsible for functional divergence. In this study, we screened the codons (Qk > 0.8) as potential sites that were crucial for functional divergence.

### Analysis of WRKY gene expansion patterns and dating the duplication events

In this study, we focused on two patterns of gene expansion: tandem duplication and segmental duplication. Tandem duplications were characterized as multiple members of this family occurring within the same or neighboring intergenic regions, where the WRKY genes were clustered together with a maximum of 10 extra genes between them [40]. Segmental duplications of each WRKY gene within the family in soybean and *Arabidopsis* genomes were searched in the PGDD (http://chibba.agtec.uga.edu/duplication/). Within the range of 100 kb, the anchors with synonymous substitution rates (Ks) values greater than 1.0 were discarded because of the risk of saturation. Assuming a molecular clock, the Ks values of duplicated genes are expected to be similar over time [88]. Therefore, we used Ks values to estimate the dates of the segmental duplication events. The mean Ks value was calculated for each pair of genes within a duplicated block and was then used to date the duplication events. The approximate date of the duplication event was then calculated using the mean Ks values (T = Ks/2λ), assuming clock-like rates (λ) of synonymous substitution of 1.5 × 10^−8^ substitutions/synonymous site/year for *Arabidopsis*[89] and 6.1 × 10^−9^ for soybean [90].

## Competing interests

The authors declare that they have no competing interests.

## Authors’ contributions

GY and HX for bioinformatics analysis and writing of the manuscript. SX and YQ for the discussion of the evolutionary pattern of WRKY genes. YL, YY, GY and YH for discussion and comments on an earlier version of the manuscript. All authors read and approved the final manuscript.

## Supplementary Material

Additional file 1 WRKY gene family in soybean. Click here for file

Additional file 2 **Schematic diagram of amino acid motifs of soybean WRKY proteins from different groups (or subgroups).** Motif analysis was performed using MEME, as described in the Methods. The grey solid line represents the corresponding WRKY protein and its length. The different-colored boxes represent different motifs and their position in each WRKY sequence. A detailed motif introduction is shown in Additional file 3. Click here for file

Additional file 3 **Schematic diagram of WRKY protein motifs.** The schematic diagram was derived from MEME. The order of motifs of WRKY proteins in the diagram was automatically generated by MEME according to scores. Click here for file

Additional file 4 **Exon/intron structures of soybean WRKY genes.** The boxes and lines represent exons and introns, respectively. The bold, dark blue lines indicate the 3’ downstream region. The WRKY genes were separated according to group or subgroup. Click here for file

Additional file 5 **Promoter analysis of the soybean WRKY gene family.** The locus names and *cis*-acting element names are listed. Click here for file

Additional file 6 Tests for positive selection among codons of WRKY genes using site models. Click here for file

Additional file 7 **Maximum likelihood estimates of the coefficient of Type-I functional divergence (θ) from pairwise comparisons between WRKY groups.** Posterior probability (PP) of the site-specific Type-I functional divergence is provided. Click here for file

Additional file 8 Estimates of the coefficient of Type-II functional divergence (θ). Click here for file

Additional file 9 **Estimates of the dates for the segmental duplication events of WRKY family in *Arabidopsis*.** Click here for file
