# Supplementary material for: The large soybean (Glycine max) WRKY TF family expanded by segmental duplication events and subsequent divergent selection among subgroups
Source: BMC Plant Biol. 2013 Oct 3;13:148. doi: 10.1186/1471-2229-13-148 (PMC3850935; doi:10.1186/1471-2229-13-148)
Supplement: Additional file 1 — WRKY gene family in soybean. [file 1471-2229-13-148-S1.docx]

**Additional File 1:** WRKY gene family in soybean.

| Gene locus | WRKY domain | | | CDS length | Gene length | Group |
| --- | --- | --- | --- | --- | --- | --- |
|  | Conserved heptapeptide | Zinc-finger type | Domain number |  |  |  |
| Glyma17g08170● | WRKYGQK/WRKYGQK | C2H2 | 2 | 1518 | 8146 | I |
| Glyma02g36510 | WRKYGQK/WRKYGQK | C2H2 | 2 | 1518 | 7638 | I |
| Glyma06g27440 | WRKYGQK/WRKYGQK | C2H2 | 2 | 1257 | 6225 | I |
| Glyma12g23950● | WRKYGQK/WRKYGQK | C2H2 | 2 | 1404 | 9173 | I |
| Glyma18g49830 | WRKYGQK/WRKYGQK | C2H2 | 2 | 1563 | 6358 | I |
| Glyma08g26230● | WRKYGQK/WRKYGQK | C2H2 | 2 | 1572 | 4709 | I |
| Glyma06g47880● | WRKYGQK/WRKYGQK | C2H2 | 2 | 2061 | 3715 | I |
| Glyma04g12830 | WRKYGQK/WRKYGQK | C2H2 | 2 | 2286 | 4718 | I |
| Glyma14g01010 | WRKYGQK/WRKYGQK | C2H2 | 2 | 1560 | 3688 | I |
| Glyma02g47650 | WRKYGQK/WRKYGQK | C2H2 | 2 | 1524 | 2991 | I |
| Glyma08g43770● | WRKYGQK/WRKYGQK | C2H2 | 2 | 1791 | 5981 | I |
| Glyma02g46690 | WRKYGQK/WRKYGQK | C2H2 | 2 | 1767 | 5787 | I |
| Glyma14g01980● | WRKYGQK/WRKYGQK | C2H2 | 2 | 1758 | 5553 | I |
| Glyma20g03410 | WRKYGQK/WRKYGQK | C2H2 | 2 | 1320 | 4610 | I |
| Glyma02g12490 | WRKYGQK/WRKYGQK | C2H2 | 2 | 1368 | 4995 | I |
| Glyma01g06550● | WRKYGQK/WRKYGQK | C2H2 | 2 | 1368 | 5227 | I |
| Glyma19g36100 | WRKYGQK/WRKYGQK | C2H2 | 2 | 1416 | 8311 | I |
| Glyma03g33380● | WRKYGQK/WRKYGQK | C2H2 | 2 | 1263 | 3444 | I |
| Glyma18g44030 | WRKYGQK/WRKYGQK | C2H2 | 2 | 1626 | 3008 | I |
| Glyma03g05220 | **WRKYGEK**/WRKYGQK | C2H2 | 2 | 1104 | 1880 | I |
| Glyma01g31920● | **WRKYGEK**/WRKYGQK | C2H2 | 2 | 1350 | 2777 | I |
| Glyma02g39870● | WRKYGQK/WRKYGQK | C2H2 | 2 | 1743 | 3376 | I |
| Glyma11g29720● | WRKYGQK/WRKYGQK | C2H2 | 2 | 1647 | 3844 | I |
| Glyma17g33920● | WRKYGQK | C2H2 | 1 | 837 | 2248 | II-a |
| Glyma14g11920 | WRKYGQK | C2H2 | 1 | 837 | 2733 | II-a |
| Glyma14g11960 | WRKYGQK | C2H2 | 1 | 858 | 1717 | II-a |
| Glyma06g06530● | WRKYGQK | C2H2 | 1 | 885 | 1768 | II-a |
| Glyma15g00570● | WRKYGQK | C2H2 | 1 | 921 | 2044 | II-a |
| Glyma13g44730 | WRKYGQK | C2H2 | 1 | 930 | 2002 | II-a |
| Glyma08g23380● | WRKYGQK | C2H2 | 1 | 942 | 2310 | II-a |
| Glyma07g02630 | WRKYGQK | C2H2 | 1 | 936 | 2320 | II-a |
| Glyma15g11680 | WRKYGQK | C2H2 | 1 | 1674 | 2648 | II-b |
| Glyma09g00820● | WRKYGQK | C2H2 | 1 | 1626 | 2474 | II-b |
| Glyma17g01490 | WRKYGQK | C2H2 | 1 | 1470 | 2529 | II-b |
| Glyma07g39250● | WRKYGQK | C2H2 | 1 | 1554 | 3307 | II-b |
| Glyma19g40950 | WRKYGQK | C2H2 | 1 | 1593 | 4782 | II-b |
| Glyma03g38360 | WRKYGQK | C2H2 | 1 | 1626 | 4184 | II-b |
| Glyma13g38630● | WRKYGQK | C2H2 | 1 | 1845 | 2851 | II-b |
| Glyma12g10350 | WRKYGQK | C2H2 | 1 | 1686 | 3111 | II-b |
| Glyma06g46420 | WRKYGQK | C2H2 | 1 | 1743 | 3192 | II-b |
| Glyma17g04710 | WRKYGQK | C2H2 | 1 | 1209 | 4721 | II-b |
| Glyma13g17800 | WRKYGQK | C2H2 | 1 | 1227 | 3514 | II-b |
| Glyma15g20990 | WRKYGQK | C2H2 | 1 | 1356 | 5590 | II-b |
| Glyma09g09400 | WRKYGQK | C2H2 | 1 | 1041 | 4995 | II-b |
| Glyma06g20300 | WRKYGQK | C2H2 | 1 | 1821 | 5089 | II-b |
| Glyma05g01280 | WRKYGQK | C2H2 | 1 | 1572 | 3826 | II-b |
| Glyma18g16170 | WRKYGQK | C2H2 | 1 | 1248 | 2571 | II-b |
| Glyma01g05050 | WRKYGQK | C2H2 | 1 | 1392 | 2622 | II-b |
| Glyma19g02440 | WRKYGQK | C2H2 | 1 | 1469 | 2907 | II-b |
| Glyma18g49140 | WRKYGQK | C2H2 | 1 | 1416 | 2944 | II-b |
| Glyma18g47350● | WRKYGQK | C2H2 | 1 | 579 | 1580 | II-c |
| Glyma09g39000 | WRKYGQK | C2H2 | 1 | 579 | 1495 | II-c |
| Glyma16g03480 | WRKYGQK | C2H2 | 1 | 528 | 2506 | II-c |
| Glyma19g26400● | WRKYGQK | C2H2 | 1 | 567 | 2452 | II-c |
| Glyma16g05880 | WRKYGQK | C2H2 | 1 | 588 | 2796 | II-c |
| Glyma08g01430● | **WRKYGEK** | C2H2 | 1 | 444 | 1209 | II-c |
| Glyma08g15210● | WRKYGQK | C2H2 | 1 | 708 | 2355 | II-c |
| Glyma06g15260 | WRKYGQK | C2H2 | 1 | 711 | 3451 | II-c |
| Glyma04g39620● | WRKYGQK | C2H2 | 1 | 369 | 2539 | II-c |
| Glyma09g37930 | WRKYGQK | C2H2 | 1 | 687 | 6014 | II-c |
| Glyma03g25770 | WRKYGQK | C2H2 | 1 | 717 | 8090 | II-c |
| Glyma08g08720 | WRKYGQK | C2H2 | 1 | 942 | 1786 | II-c |
| Glyma05g25770 | WRKYGQK | C2H2 | 1 | 1077 | 2371 | II-c |
| Glyma14g03280 | WRKYGQK | C2H2 | 1 | 1017 | 2477 | II-c |
| Glyma02g45530 | WRKYGQK | C2H2 | 1 | 945 | 2467 | II-c |
| Glyma02g12830● | WRKYGQK | C2H2 | 1 | 882 | 7280 | II-c |
| Glyma01g06870● | WRKYGQK | C2H2 | 1 | 894 | 3658 | II-c |
| Glyma08g15050● | **WRKYGKK** | C2H2 | 1 | 555 | 3297 | II-c |
| Glyma05g31800 | **WRKYGKK** | C2H2 | 1 | 567 | 3102 | II-c |
| Glyma06g15220● | **WRKYGKK** | C2H2 | 1 | 591 | 3408 | II-c |
| Glyma04g39650● | **WRKYGKK** | C2H2 | 1 | 621 | 2888 | II-c |
| Glyma04g05700 | **WRKYGKK** | C2H2 | 1 | 486 | 1114 | II-c |
| Glyma17g34210 | **WRKYGKK** | C2H2 | 1 | 570 | 1479 | II-c |
| Glyma14g11440 | **WRKYGKK** | C2H2 | 1 | 450 | 1308 | II-c |
| Glyma15g14860 | WRKYGQK | C2H2 | 1 | 1068 | 2774 | II-c |
| Glyma09g03900● | WRKYGQK | C2H2 | 1 | 996 | 1996 | II-c |
| Glyma17g03950 | WRKYGQK | C2H2 | 1 | 1197 | 3101 | II-c |
| Glyma07g36640 | WRKYGQK | C2H2 | 1 | 1128 | 2059 | II-c |
| Glyma19g40560 | WRKYGQK | C2H2 | 1 | 873 | 1525 | II-c |
| Glyma03g37940● | WRKYGQK | C2H2 | 1 | 864 | 1958 | II-c |
| Glyma10g01450● | WRKYGQK | C2H2 | 1 | 972 | 2129 | II-c |
| Glyma02g01420 | WRKYGQK | C2H2 | 1 | 963 | 2140 | II-c |
| Glyma17g29190 | WRKYGQK | C2H2 | 1 | 951 | 2987 | II-d |
| Glyma14g17730● | WRKYGQK | C2H2 | 1 | 951 | 2931 | II-d |
| Glyma04g08060● | WRKYGQK | C2H2 | 1 | 840 | 2043 | II-d |
| Glyma06g08120● | WRKYGQK | C2H2 | 1 | 903 | 1819 | II-d |
| Glyma17g06450● | WRKYGQK | C2H2 | 1 | 963 | 1661 | II-d |
| Glyma13g00380● | WRKYGQK | C2H2 | 1 | 975 | 1616 | II-d |
| Glyma15g18250● | WRKYGQK | C2H2 | 1 | 882 | 2021 | II-d |
| Glyma09g06980● | WRKYGQK | C2H2 | 1 | 891 | 2131 | II-d |
| Glyma10g03820 | WRKYGQK | C2H2 | 1 | 1179 | 2899 | II-d |
| Glyma02g15920● | WRKYGQK | C2H2 | 1 | 1068 | 2873 | II-d |
| Glyma03g31630● | WRKYGQK | C2H2 | 1 | 1026 | 2080 | II-d |
| Glyma17g18480 | WRKYGQK | C2H2 | 1 | 999 | 2042 | II-d |
| Glyma05g20710● | WRKYGQK | C2H2 | 1 | 1005 | 2135 | II-d |
| Glyma11g05650● | WRKYGQK | C2H2 | 1 | 966 | 1663 | II-d |
| Glyma01g39600 | WRKYGQK | C2H2 | 1 | 966 | 1911 | II-d |

**S1** continued

| Gene locus | WRKY domain | | | CDS length | Gene length | Group |
| --- | --- | --- | --- | --- | --- | --- |
|  | Conserved heptapeptide | Zinc-finger type | Domain number |  |  |  |
| Glyma15g14370 | WRKYGQK | C2H2 | 1 | 933 | 3175 | II-e |
| Glyma09g03450 | WRKYGQK | C2H2 | 1 | 1353 | 3027 | II-e |
| Glyma05g25330 | WRKYGQK | C2H2 | 1 | 897 | 3597 | II-e |
| Glyma08g08340 | WRKYGQK | C2H2 | 1 | 1290 | 3933 | II-e |
| Glyma08g12460● | WRKYGQK | C2H2 | 1 | 786 | 1939 | II-e |
| Glyma05g29310 | WRKYGQK | C2H2 | 1 | 768 | 1824 | II-e |
| Glyma13g36540● | WRKYGQK | C2H2 | 1 | 798 | 2798 | II-e |
| Glyma12g33990● | WRKYGQK | C2H2 | 1 | 792 | 2759 | II-e |
| Glyma08g02160 | WRKYGQK | C2H2 | 1 | 840 | 1544 | II-e |
| Glyma05g37390 | WRKYGQK | C2H2 | 1 | 798 | 1475 | II-e |
| Glyma18g47300● | WRKYGQK | C2H2 | 1 | 1056 | 1620 | II-e |
| Glyma09g39040 | WRKYGQK | C2H2 | 1 | 1047 | 2076 | II-e |
| Glyma16g03570 | WRKYGQK | C2H2 | 1 | 1008 | 1990 | II-e |
| Glyma09g24080 | WRKYGQK | C2H2 | 1 | 867 | 1452 | II-e |
| Glyma20g30290 | WRKYGQK | C2H2 | 1 | 969 | 1262 | II-e |
| Glyma10g37460● | WRKYGQK | C2H2 | 1 | 837 | 1594 | II-e |
| Glyma19g40470● | WRKYGQK | C2H2 | 1 | 795 | 1887 | II-e |
| Glyma03g37870● | WRKYGQK | C2H2 | 1 | 762 | 1945 | II-e |
| Glyma13g34280 | WRKYGQK | C2HC | 1 | 497 | 1578 | III |
| Glyma03g00460 | WRKYGQK | C2HC | 1 | 747 | 1801 | III |
| Glyma18g44560● | WRKYGQK | C2HC | 1 | 900 | 2009 | III |
| Glyma09g41050 | WRKYGQK | C2HC | 1 | 903 | 1982 | III |
| Glyma14g36430 | WRKYGQK | C2HC | 1 | 696 | 3489 | III |
| Glyma06g14720 | WRKYGQK | C2HC | 1 | 960 | 2734 | III |
| Glyma04g40130● | WRKYGQK | C2HC | 1 | 954 | 2555 | III |
| Glyma08g02580● | WRKYGQK | C2HC | 1 | 1080 | 2224 | III |
| Glyma05g36970 | WRKYGQK | C2HC | 1 | 1092 | 2983 | III |
| Glyma01g43420● | WRKYGQK | C2HC | 1 | 969 | 1447 | III |
| Glyma06g13090 | WRKYGQK | C2HC | 1 | 1095 | 2130 | III |
| Glyma19g44380 | WRKYGQK | C2HC | 1 | 1089 | 2622 | III |
| Glyma03g41750● | WRKYGQK | C2HC | 1 | 1089 | 2578 | III |
| Glyma16g02960● | WRKYGQK | C2HC | 1 | 1122 | 3244 | III |
| Glyma07g06320● | WRKYGQK | C2HC | 1 | 1110 | 2898 | III |
| Glyma04g40120 | WRKYGQK | C2HC | 1 | 501 | 1243 | III |
| Glyma18g39970 | WRKYGQK | C2H2 | 1 | 864 | 3456 | NG |
| Glyma14g37960 | **WRKYEDK** | C2H2 | 1 | 999 | 1148 | NG |

NG: none of group, *Glyma18g39970* and *Glyma14g37960* were not assigned to any group and existed alone. The variants of conserved WRKYGQK peptide are shown in bold. Black circle represent that these genes had been identified by zhou YQ at el. (2008).
