# Supplementary figures and images for: The large soybean (Glycine max) WRKY TF family expanded by segmental duplication events and subsequent divergent selection among subgroups
Source: BMC Plant Biol. 2013 Oct 3;13:148. doi: 10.1186/1471-2229-13-148 (PMC3850935; doi:10.1186/1471-2229-13-148)

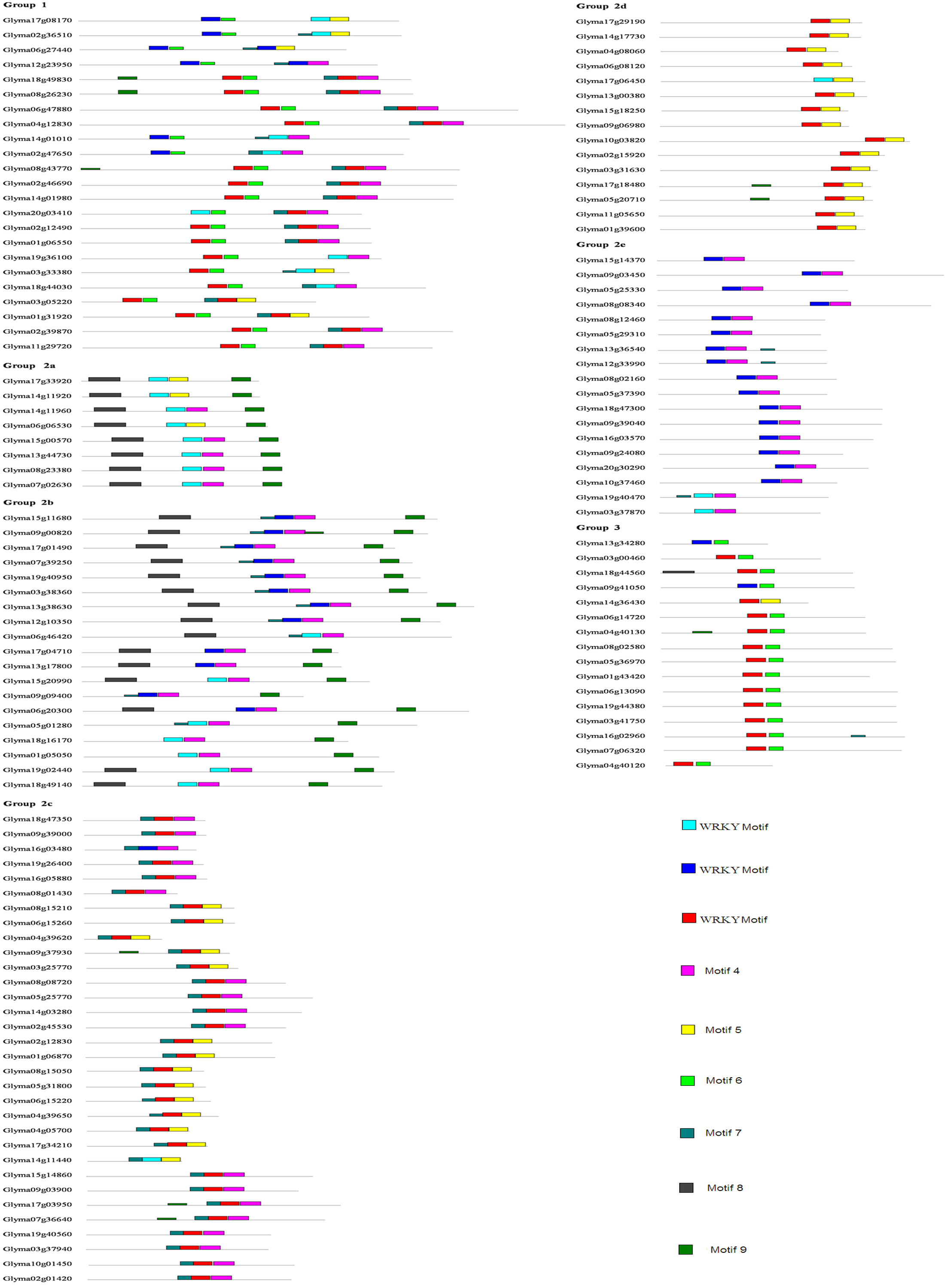

Supplement: Additional file 2 — Schematic diagram of amino acid motifs of soybean WRKY proteins from different groups (or subgroups). Motif analysis was performed using MEME, as described in the Methods. The grey solid line represents the corresponding WRKY protein and its length. The different-colored boxes represent different motifs and their position in each WRKY sequence. A detailed motif introduction is shown in Additional file 3. [file 1471-2229-13-148-S2.tiff]

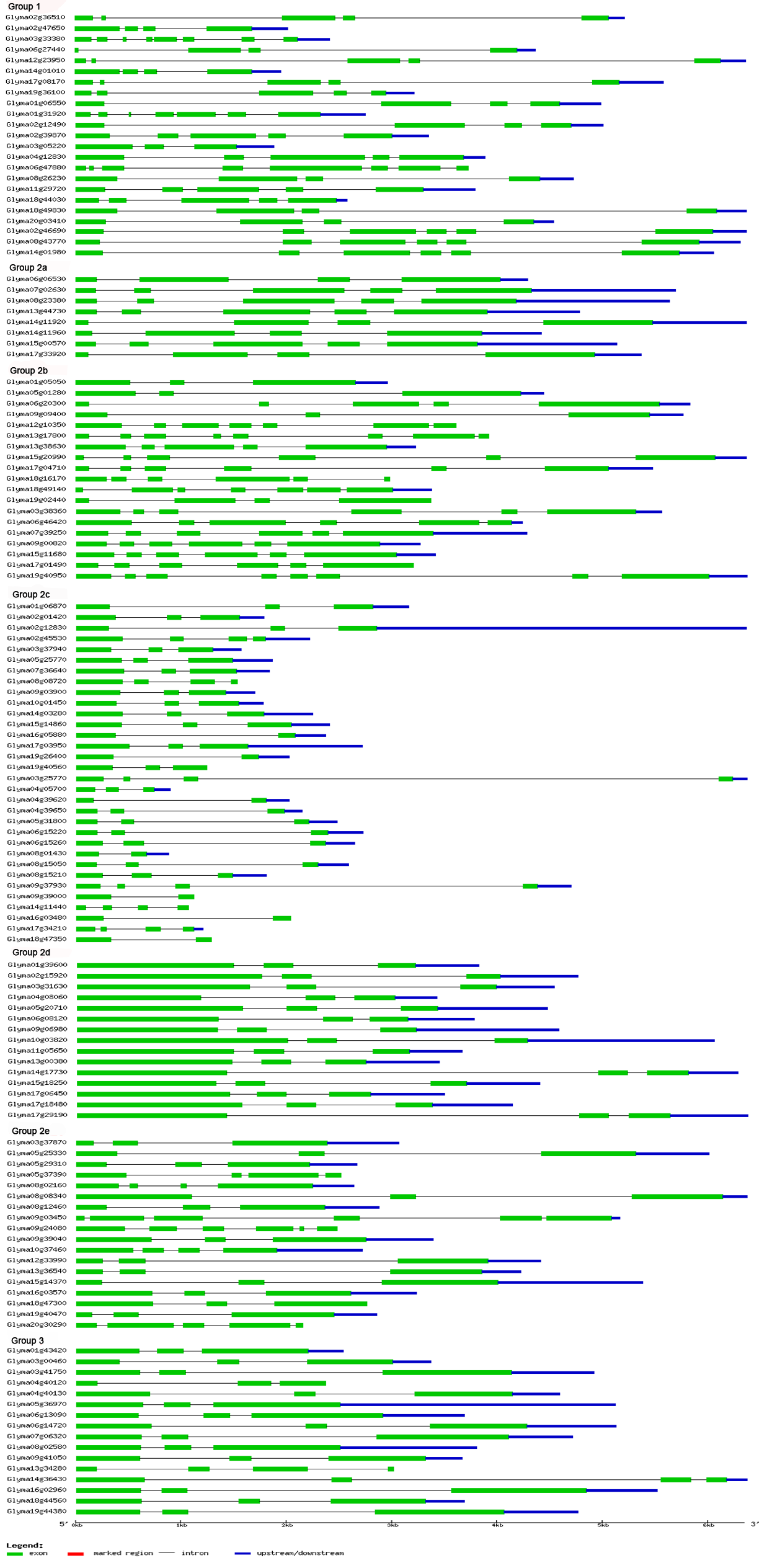

Supplement: Additional file 4 — Exon/intron structures of soybean WRKY genes. The boxes and lines represent exons and introns, respectively. The bold, dark blue lines indicate the 3’ downstream region. The WRKY genes were separated according to group or subgroup. [file 1471-2229-13-148-S4.tiff]
