# Supplementary material for: The large soybean (Glycine max) WRKY TF family expanded by segmental duplication events and subsequent divergent selection among subgroups
Source: BMC Plant Biol. 2013 Oct 3;13:148. doi: 10.1186/1471-2229-13-148 (PMC3850935; doi:10.1186/1471-2229-13-148)

**Additional File 3:** Schematic diagram of WRKY protein motifs.

The schematic diagram was derived from MEME. The order of motifs of WRKY proteins in the diagram was automatically generated by MEME according to scores.


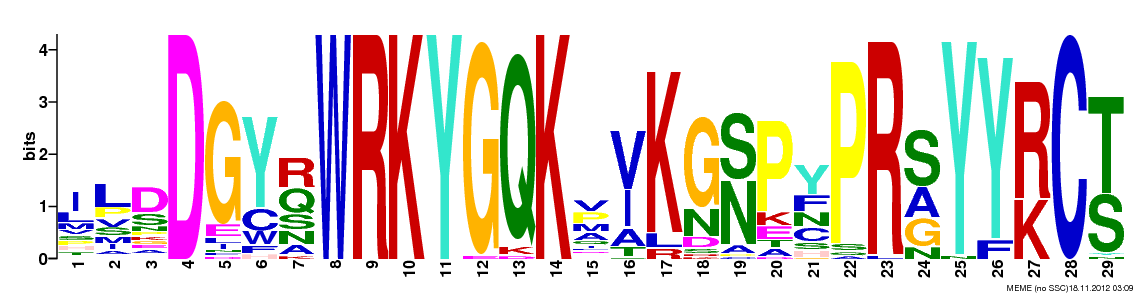


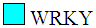


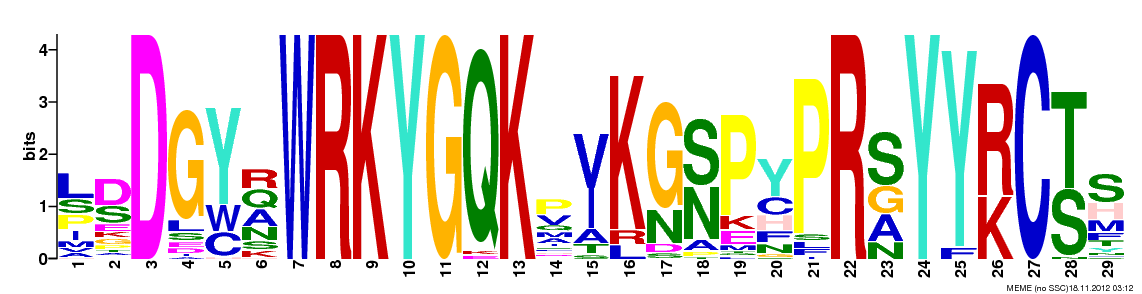


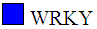


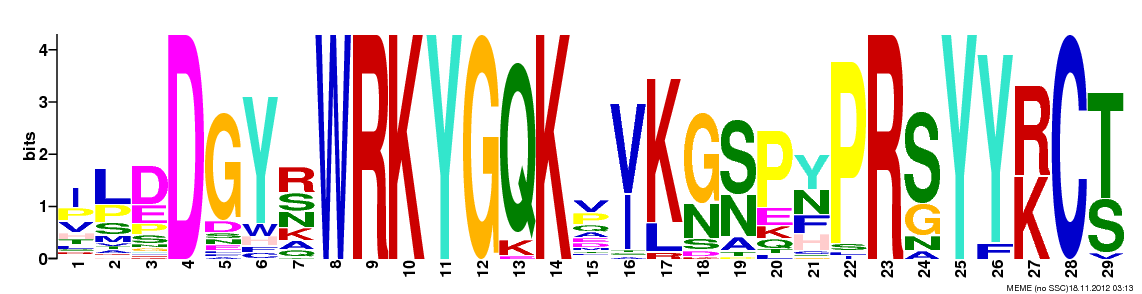


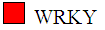


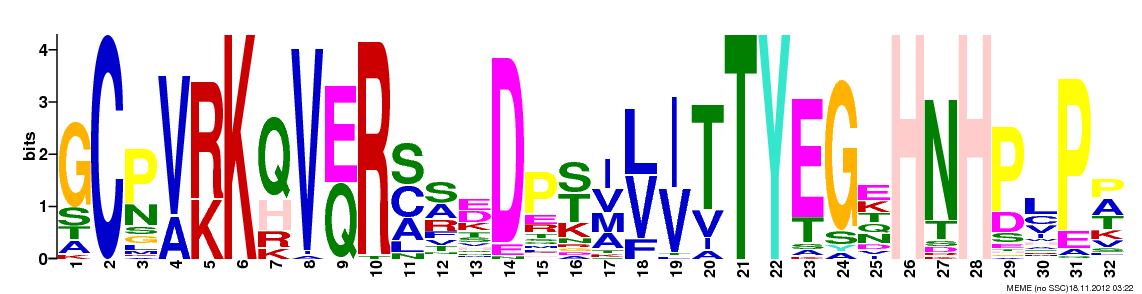


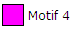


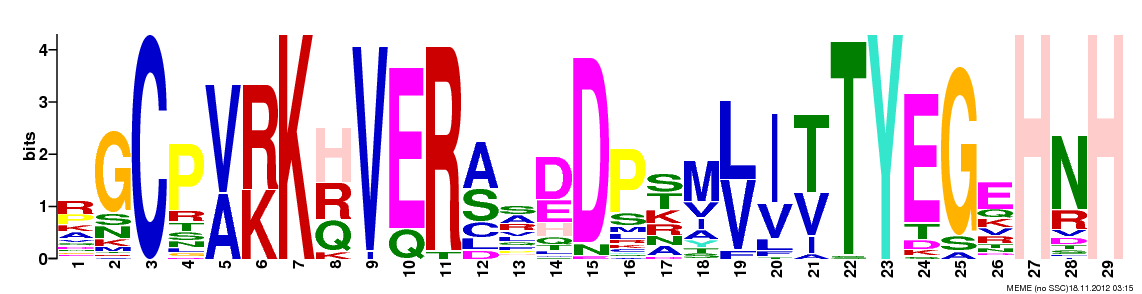


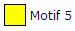


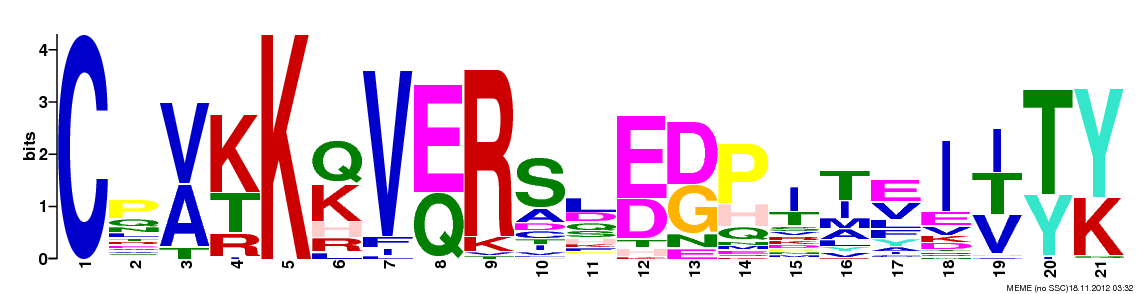


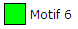


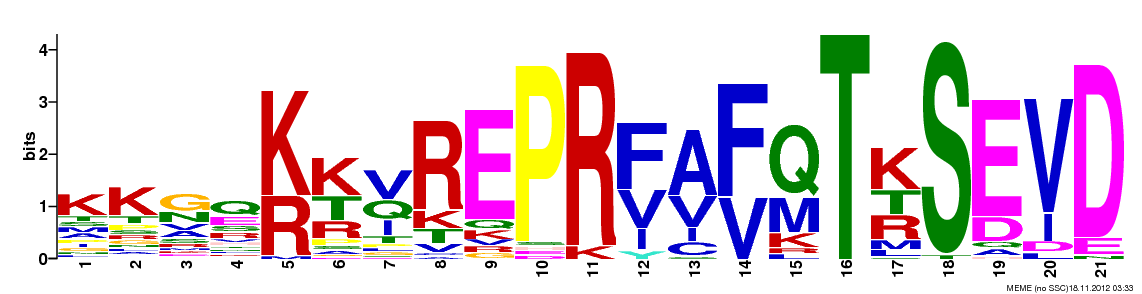


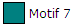


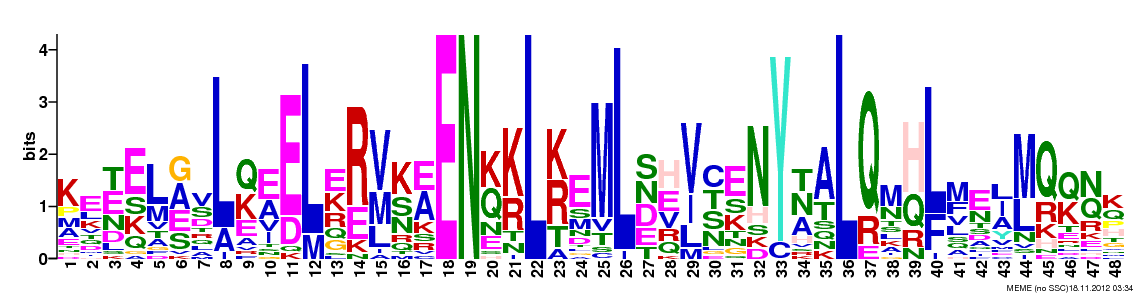


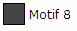


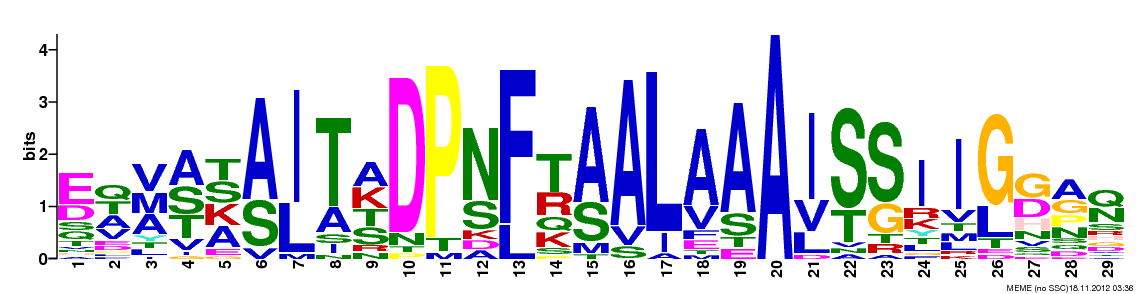


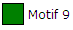

Supplement: Additional file 3 — Schematic diagram of WRKY protein motifs. The schematic diagram was derived from MEME. The order of motifs of WRKY proteins in the diagram was automatically generated by MEME according to scores. [file 1471-2229-13-148-S3.docx]
