# Supplementary material for: The large soybean (Glycine max) WRKY TF family expanded by segmental duplication events and subsequent divergent selection among subgroups
Source: BMC Plant Biol. 2013 Oct 3;13:148. doi: 10.1186/1471-2229-13-148 (PMC3850935; doi:10.1186/1471-2229-13-148)
Supplement: Additional file 6 — Tests for positive selection among codons of WRKY genes using site models. [file 1471-2229-13-148-S6.docx]

**Additional File 6**. Tests for positive selection among codons of WRKY genes using site models.

Site Model

| Model | lnL | Estimates of parameter ^a^ | 2ΔlnL | positive selection sites ^b^ |
| --- | --- | --- | --- | --- |
|  |  |  |  |  |
| M0(one-ratio) | -8608.409 | ω=0.056 | 335.95(M3vsM0)** | Not allowed |
| M3(discrete) | -8272.457 | p_0_=0.277 ω_0_=0.002  p_1_=0.410 ω_1_=0.035  p_2_=0.312 ω_2_=0.153 |  | None |
| M7(beta) | -8253.022 | p=0.452 q=6.290 | 1944.18(M7vsM8)** | Not allowed |
| M8(beta & ω) | -10197.202 | p_0_=0.99999 p=0.467  q= 1.991 p_1_=0.00001  ω=2.638 |  | 274V*, 278P**,  287Q**, 291N** |
|  |  |  |  |  |

Note: * p < 0.05 and ** p < 0.01 (*x*^2^ test).

a ω was estimated under model M0,M3,M7, and M8; p and q are the parameters of the beta distribution.

b The number of amino acid sites estimated to have undergone positive selection.
