# Supplementary material for: The large soybean (Glycine max) WRKY TF family expanded by segmental duplication events and subsequent divergent selection among subgroups
Source: BMC Plant Biol. 2013 Oct 3;13:148. doi: 10.1186/1471-2229-13-148 (PMC3850935; doi:10.1186/1471-2229-13-148)
Supplement: Additional file 7 — Maximum likelihood estimates of the coefficient of Type-I functional divergence (θ) from pairwise comparisons between WRKY groups. Posterior probability (PP) of the site-specific Type-I functional divergence is provided. [file 1471-2229-13-148-S7.docx]

**Additional File 7**. Maximum likelihood estimates of the coefficient of Type-I functional divergence (θ) from pairwise comparisons between WRKY groups. Posterior probability (PP) of the site-specific Type-I functional divergence is provided.

GU99

| Group | θ _ML_ | θ _SE_ | θ _LRT_ | Q k | P |
| --- | --- | --- | --- | --- | --- |
|  |  |  |  |  |  |
| Group 2d/Group2e | 0.027 | 0.193 | 0.019 | None |  |
| Group 2d/Group 3 | 0.021 | 0.310 | 0.005 | None |  |
| Group 2d/Group2b | 0.390 | 0.181 | 4.639 | 289D | P<0.05 |
| Group 2d/Group2a | 0.352 | 0.289 | 1.486 | None |  |
| Group 2d/Group 2c | 0.395 | 0.154 | 6.612 | 246V, 289D | P<0.05 |
| Group 2d/Group 1 | 0.150 | 0.233 | 0.411 | None |  |
| Group 2e/Group 3 | 0.112 | 0.142 | 0.621 | None |  |
| Group 2e/Group2b | 0.618 | 0.133 | 21.553 | 248E, 258P, 259I, 272S,  275R, 276G, 278P, 298E,  299G, 302R | P<0.01 |
| Group 2e/Group 2a | 0.371 | 0.187 | 3.923 | 276G | P<0.05 |
| Group 2e/Group 2c | 0.466 | 0.096 | 23.509 | 248E, 258P, 262S, 263P,  264Y, 275R, 276G, 295V,  299G | P<0.01 |
| Group 2e/Group 1 | 0.372 | 0.118 | 10.019 | 248E, 264Y, 275R , 299G | P<0.01 |
| Group 3/Group 2b | 0.590 | 0.169 | 12.239 | 250S, 261G, 264Y, 278P,  288D, 295V, 298E | P<0.01 |
| Group 3/Group 2a | 0.319 | 0.262 | 1.487 | None |  |
| Group 3/Group 2c | 0.311 | 0.112 | 7.713 | 293L, 295V, 302R | P<0.01 |
| Group 3/Group 1 | 0.201 | 0.091 | 4.900 | 288D | P<0.05 |
| Group 2b/Group 2c | 0.327 | 0.088 | 13.739 | 246V, 259I, 264Y, 278P,  302R | P<0.01 |
| Group 2b/Group 1 | 0.071 | 0.131 | 0.294 | None |  |
| Group 2a/Group 2c | 0.469 | 0.167 | 7.857 | 275R, 295V | P<0.01 |
| Group 2a/Group 1 | 0.110 | 0.213 | 0.270 | None |  |
| Group 2c/Group 1 | 0.064 | 0.118 | 0.293 | None |  |
|  |  |  |  |  |  |

Note: *x*^2^ test was introduced to examined the data with statistically significant difference.
