# Supplementary material for: The large soybean (Glycine max) WRKY TF family expanded by segmental duplication events and subsequent divergent selection among subgroups
Source: BMC Plant Biol. 2013 Oct 3;13:148. doi: 10.1186/1471-2229-13-148 (PMC3850935; doi:10.1186/1471-2229-13-148)
Supplement: Additional file 8 — Estimates of the coefficient of Type-II functional divergence (θ). [file 1471-2229-13-148-S8.docx]

**Additional File 8.** Estimates of the coefficient of Type-II functional divergence (θ).

| Group | θ-II | θ _SE_ | Q k | No. of sites |
| --- | --- | --- | --- | --- |
| Group 2d/Group 2e | 0.033 | 0.124 | 250S, 282H, 286A, 298E, | 4 |
| Group 2d/Group 3 | 0.050 | 0.191 | 246V, 248E, 258P, 260K,  262S, 263P, 275R, 280R,  282H, 284E, 287Q, 294I,  298E, 302R, 303H | 15 |
| Group 2d/Group 2b | 0.246 | 0.111 | 248E, 249Y, 250S, 258P,  262S, 264Y, 267G, 270K,  275R, 276G, 279A, 282H,  284E, 286A, 287Q, 295V,  300E, 302R, | 18 |
| Group 2d/Group 2a | 0.307 | 0.119 | 246V, 248E, 250S, 258P,  259I, 260K, 261G, 262S,  264Y, 267G, 269Y, 279A,  270K, 275R, 280R, 282H,  284E, 286A, 287Q, 292M,  295V, 302R | 22 |
| Group 2d/Group 2c | 0.234 | 0.148 | 246V◆, 249Y, 250S, 258P,  259I, 264Y, 267G, 270K,  272S, 279A, 280R, 282H,  293L, 295V, 300E, 302R | 16 |
| Group 2d/Group 1 | 0.152 | 0.151 | 246V, 248E, 250S, 258P,  264Y, 275R,288D, 291N,  295V, 300E, 302R | 11 |
| Group 2e/Group 2b | 0.288 | 0.127 | 248E◆, 249Y, 250S, 258P◆,  262S, 264Y, 267G, 274V,  275R◆, 276G◆, 279A, 284E,  287Q, 288D, 295V, 298E◆,  300E | 17 |
| Group 2e/Group 2a | 0.282 | 0.139 | 246V, 248E, 249Y, 250S,  258P, 259I, 261G, 262S,  264Y, 267G, 269Y, 274V,  275R, 282H, 284E, 287Q,  288D, 295V, 298E | 19 |
| Group 2e/Group 2c | 0.188 | 0.164 | 246V, 248E◆, 250S, 258P◆,  264Y◆, 274V, 275R◆,278P,  282H, 286A, 287Q, 295V◆,  298E, 300E | 14 |
| Group 2e/Group 1 | 0.203 | 0.161 | 246V, 248E◆, 250S, 258P,  264Y◆, 275R◆, 282H, 286A, 287Q, 288D, 291N, 295V,  298E, 300E, 302R | 15 |
| Group 3/Group 2b | 0.163 | 0.176 | 246V, 249Y, 258P, 260K,  262S, 263P, 264Y◆, 267G,  276G, 280R, 287Q, 294I,  295V◆, 298E◆, 303H | 15 |
| Group 3/Group 2c | 0.143 | 0.208 | 250S, 260K, 262S, 263P,  264Y, 268P, 274V, 275R,  280R, 282H 284E, 286A,  287Q,294I, 295V◆, 298E,  300E, 303H | 18 |
| Group 3/Group 1 | 0.051 | 0.209 | 250S, 258P, 300E, 260K ,  262S, 263P, 264Y,274V,  275R, 280R, 282H, 286A,  284E, 287Q, 288D◆, 291N,  294I, 298E, 295V, 302R,  303H | 21 |
| Group 2b/Group 2a | 0.003 | 0.147 | 249Y, 261G | 2 |
| Group 2b/Group 2c | 0.086 | 0.157 | 246V◆, 249Y, 250S, 261G,  264Y◆, 267G, 276G, 286A,  282H, 287Q, 300E | 11 |
| Group 2b/Group 1 | 0.090 | 0.162 | 246V, 249Y, 250S, 264Y,  267G, 274V, 275R, 276G,  282H, 284E, 286A, 287Q,  288D, 291N,300E, 302R | 16 |
| Group 2a/Group 2c | 0.097 | 0.175 | 246V, 250S, 259I, 261G,  267G, 284E, 286A, 300E | 8 |
| Group 2a/Group 1 | 0.097 | 0.175 | 246V, 250S, 259I, 261G,  264Y, 267G, 275R, 284E,  286A, 287Q, 291N,300E,  302R, | 13 |

Note: Black rhombus indicated that amino acid residues presented both of Type-I and Type-II functional divergence between pairwise comparisons of corresponding groups or subgroups in soybean.
