# Supplementary material for: The large soybean (Glycine max) WRKY TF family expanded by segmental duplication events and subsequent divergent selection among subgroups
Source: BMC Plant Biol. 2013 Oct 3;13:148. doi: 10.1186/1471-2229-13-148 (PMC3850935; doi:10.1186/1471-2229-13-148)
Supplement: Additional file 9 — Estimates of the dates for the segmental duplication events of WRKY family in Arabidopsis. [file 1471-2229-13-148-S9.docx]

**Additional File 9.** Estimates of the dates for the segmental duplication events of WRKY family in *Arabidopsis*.

| Segment pairs | Number of  anchors | KS  (mean±s.d.) | Estimated  time (mya) |
| --- | --- | --- | --- |
| At1g13960 & At2g03340 | 9 | 0.78±0.14 | 26 |
| At1g64000 & At5g41570 | 10 | 0.72±0.11 | 24 |
| At2g24570 & At4g31550 | 8 | 0.74±0.10 | 25 |
| At2g25000 & At4g31800 | 4 | 0.80±0.10 | 27 |
| At2g47260 & At3g62340 | 9 | 0.75±0.15 | 25 |
| At4g11070 & At4g23810 | 8 | 0.76±0.13 | 25 |

Abbreviation: mya, million years ago.
